# Variations in the fuel structure control the rate of the back and forth motions of a chemically fuelled molecular switch†
†This work is dedicated to Professor Carlo Galli.
‡
‡Electronic supplementary information (ESI) available: NMR of acids **2** (X = Cl, H, CH_3_ and OCH_3_), ^1^H NMR monitoring of the decarboxylation of acids **2** (X = Cl, CH_3_ and OCH_3_) promoted by Et_3_N or catenand **1** and ^1^H NMR of **1**H^+^CF_3_CO_2_^–^. See DOI: 10.1039/c7sc04123c


**DOI:** 10.1039/c7sc04123c

**Published:** 2017-10-18

**Authors:** Chiara Biagini, Simone Albano, Rachele Caruso, Luigi Mandolini, José Augusto Berrocal, Stefano Di Stefano

**Affiliations:** a Dipartimento di Chimica and Istituto CNR di Metodologie Chimiche-IMC , Sezione Meccanismi di Reazione c/o Dipartimento di Chimica , Università degli Studi di Roma “La Sapienza” , P.le A. Moro 5 , 00185 Rome , Italy . Email: stefano.distefano@uniroma1.it; b Institute for Complex Molecular Systems , Eindhoven University of Technology , P.O. Box 513 , 5600 MB Eindhoven , The Netherlands

## Abstract

This work deals with the use of 2-cyano-2-arylpropanoic acids as chemical fuels for an acid–base operated molecular switch that consists of a Sauvage-type catenand composed of two identical macrocycles incorporating a phenanthroline unit. When used as a base promoter of the decarboxylation of propanoic acid derivatives, the switch undergoes large amplitude motion from the neutral catenand to a protonated catenate and back again to the neutral state. The rate of back proton transfer, which determines the rate of the overall process, was markedly affected by *para*-substituents in the order Cl > H > CH_3_ > OCH_3_ (*ρ* = +5.2). Thus, the time required to complete a full cycle was almost two days for the OCH_3_ derivative and dropped to a few minutes for the Cl derivative. These results show for the first time that the rate of operation of a molecular switch can be regulated by variations in the fuel structure.

## Abstract 2

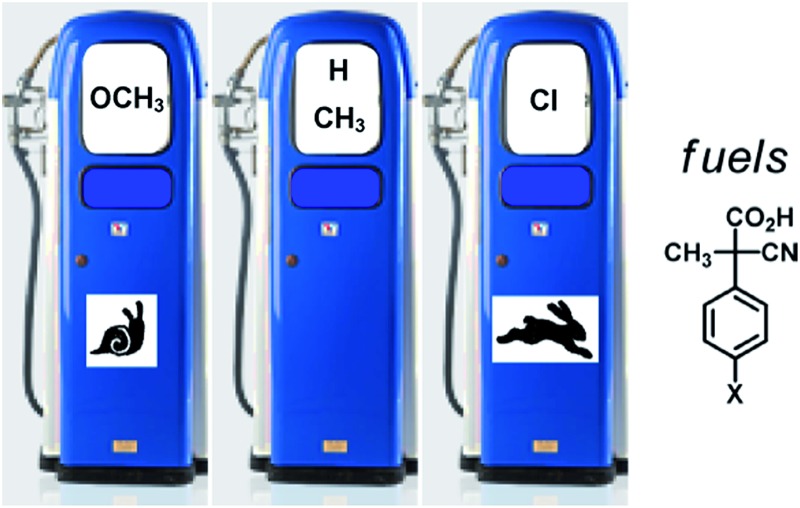
Moderate variations in the fuel structure cause large changes in the rate of the back and forth motions experienced by a chemically fuelled catenane-based switch.

## Introduction

Great effort has been devoted in recent years to the development of molecular machines.^1^ Prototypal examples of molecular machines are mechanically interlocked systems, such as rotaxanes and catenanes, characterized by various levels of structural complexity. The distinctive feature of these systems is the ability to perform back and forth motions, *i.e.* a series of two or more roto-translations during which the machine varies from an initial state A to one or more intermediate states, B′, B′′,..., and eventually reverts back to the initial state A.

The transition between states A and B usually requires the sequential addition of a fuel and an anti-fuel, such as an acid and a base,^2^ a reductant and an oxidant,^3^ irradiation with light of different wavelengths^4^ and the like. Systems in which only one stimulus is required to guarantee the operation of a back and forth motion are rare. Early examples were reported by Leigh and Wurpel *et al.*5*a* and Balzani, Credi and Stoddart *et al.*,5*b* who used light irradiation to induce cyclic motion in a rotaxane structure.

**Scheme 1:**
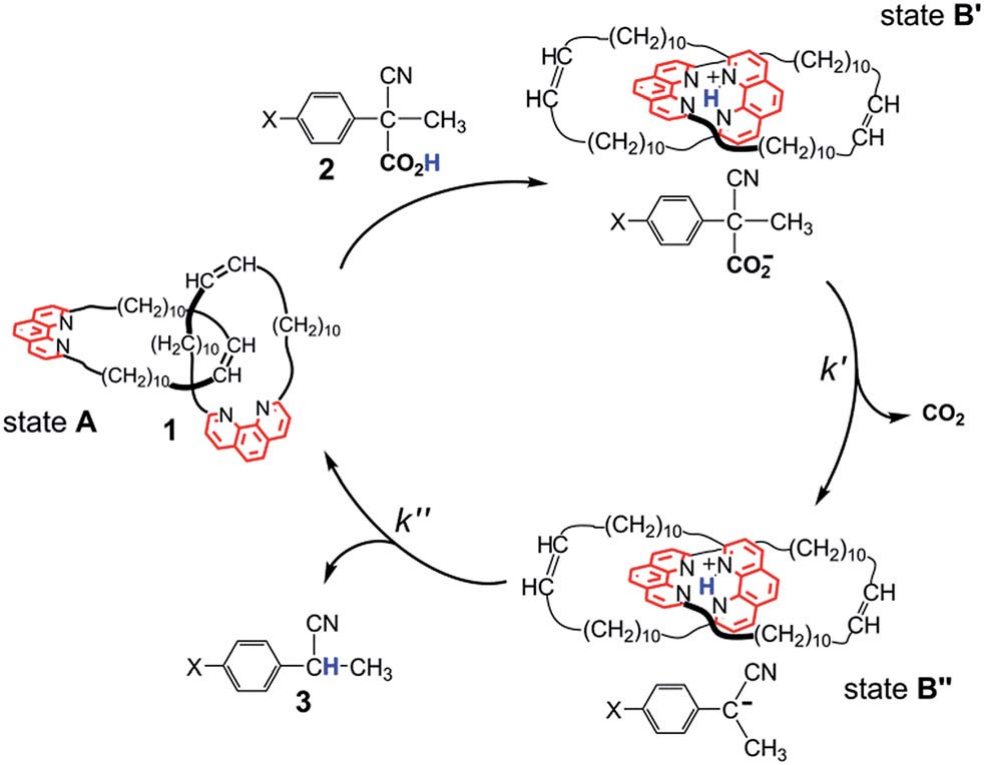
Switching motions of catenane **1** fuelled by acid **2** (X = H). Fast and quantitative proton transfer (step **A** → **B′**) fixes the contact segments between the interlocked macrocycles in the region of the phenanthroline nitrogen atoms. Loss of CO_2_ (step **B′** → **B′′**), followed by slower back proton transfer from **1**H^+^ to its counteranion, restores the catenane to its original form (step **B′′** → **A**).

It is only lately that molecular machines operated by the irreversible reaction of a single chemical fuel have been reported by us^6^ and other workers.^7^ Our system is described in Scheme 1. The Sauvage-type [2]catenand **1**, composed of two identical macrocycles incorporating 1,10-phenanthroline units, performs fuelled switching between states **A** and **B′**–**B′′**. While **B′** and **B′′** are well-defined states with the two phenanthroline subunits tightly held together by interactions with the shared proton, state **A** is not co-conformationally well-defined. The fuel, 2-cyano-2-phenylpropanoic acid (**2**, X = H) undergoes base-promoted quantitative decarboxylation *via* a carbanion intermediate, which is rapidly formed (step **B′** → **B′′**) and slowly transformed (step **B′′** → **A**) to the “waste” product 2-phenylpropanonitrile (**3**, X = H) by back proton transfer.

Since the stability and reactivity of carbanions are strongly structure dependent,^8^ we reasoned that the rate of the back and forth motion could be conveniently controlled by structural variations of the acid fuel. Here we report an assessment of the influence of the substituents in the *para* positions of 2-cyano-2-(*p*-chlorophenyl)propanoic acid (**2**, X = Cl), 2-cyano-2-(*p*-methylphenyl)propanoic acid (**2**, X = CH_3_) and 2-cyano-2-(*p*-methoxyphenyl)propanoic acid (**2**, X = OCH_3_) on the rate of the fuelled switching of catenand **1**.

## Results and discussion

**Scheme 2:**
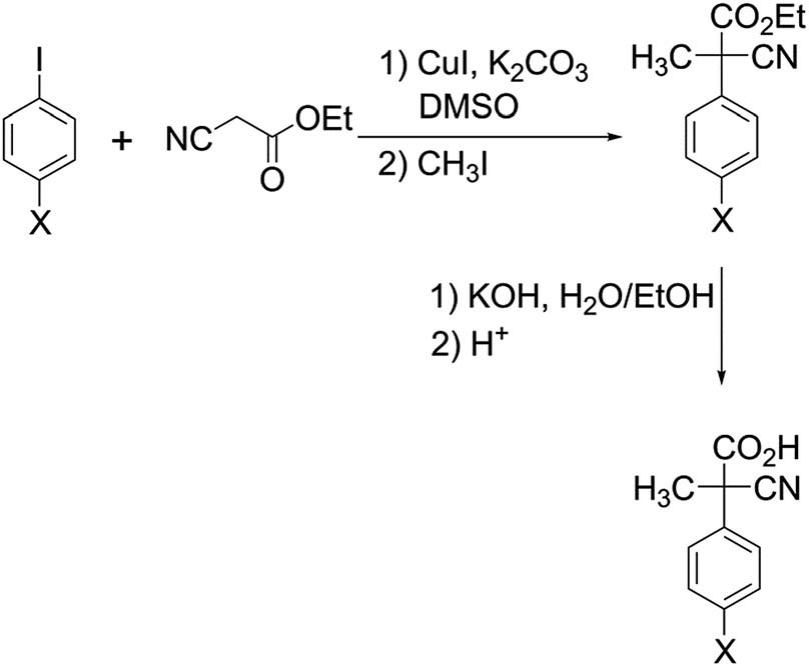
Preparation of acids **2** (X = Cl, CH_3_ and OCH_3_).

Compounds **2** (X = Cl, CH_3_ and OCH_3_) were prepared according to the same procedure adopted for the preparation of the parent acid **2** (X = H) (Scheme 2).^6^

In a set of control experiments aimed at assessing the effect of the substituents on the rate of rupture of the R–CO_2_^–^ bond, the Et_3_N promoted decarboxylation of the acid derivatives was monitored by ^1^H NMR spectroscopy in CD_2_Cl_2_ at 25 °C for comparison with the corresponding reaction of the parent acid **2** (X = H). For the latter it has been established^6^ that fast and quantitative proton transfer from acid **2** (X = H) to Et_3_N generates a hydrogen bonded ion pair§
§The idea that the ion pair is strongly hydrogen bonded is consistent, *inter alia*, with the finding that the time required to complete the decarboxylation of **2** (X = H) dropped from about 3 h to 10 min when Et_3_N was replaced by the proton sponge 1,8-bis(dimethylamino)naphthalene (see ref. 6). The protonated proton sponge, where the proton is covalently bound to one nitrogen atom and hydrogen bonded to the other, stabilizes the carboxylate counterion much less effectively than Et_3_NH^+^. RCO_2_^–^···HNEt_3_^+^. The rate-determining liberation of CO_2_ leads to a carbanion intermediate R^–^, presumably ion paired to Et_3_NH^+^.¶
¶Because of the low polarity of dichloromethane (*ε* = 9.08) the concentration of free ions is most likely negligibly small. Fast proton transfer from Et_3_NH^+^ to R^–^ completes the reaction and restores the neutral form of Et_3_N.

Decarboxylation proceeded smoothly with the other substituents as well and afforded a quantitative yield of the decarboxylated product **3** in all cases (see ESI, Fig. S1–S3‡). The half-lives of 14, 36, 204 and 332 min, determined from the ^1^H NMR spectra, for the sequence X = Cl, H, CH_3_ and OCH_3_, respectively, are in line with expectations for a reaction in which a fractional negative charge develops on the benzylic carbon atom in the transition state. A Hammett plot of log *k*_rel_*vs. σ*_p_ gave *ρ* = 2.9 (*r* = 0.98) (see ESI, Fig. S4‡).

Let us now turn to the use of catenand **1** as a base promoter of decarboxylation. A marked difference from the Et_3_N promoted reaction was expected, because evidence was obtained that the back proton transfer to the carbanion intermediate (Scheme 1, step **B′′** → **A**) becomes the slow step in the decarboxylation of the parent acid **2** (X = H).^6^^1^H NMR spectra of a 1 : 1 mixture of **1** and **2** (X = H) (see ESI, Fig. S6‡) revealed the presence of a number of signals belonging to a reaction intermediate which was thought to be a tight ion pair composed of the protonated catenate **1**H^+^ and the carbanion produced in step **B′** → **B′′**. Proton transfer from **1**H^+^ to its counteranion restores the catenane to its original form (step **B′′** → **A**). The exceptional slowness of the proton removal from the tetrahedral cavity defined by the four N atoms‖
‖When the counteranion of **1**H^+^ is such a weakly interacting anion as CF_3_CO_2_^–^, the equivalence of the two phenanthroline units in the ^1^H NMR spectrum (see ESI, Fig. S9‡) points to a symmetrical species in which scrambling of the proton among the nitrogen atoms is fast on the ^1^H NMR timescale. was ascribed to steric hindrance.^6^

**Fig. 1:**
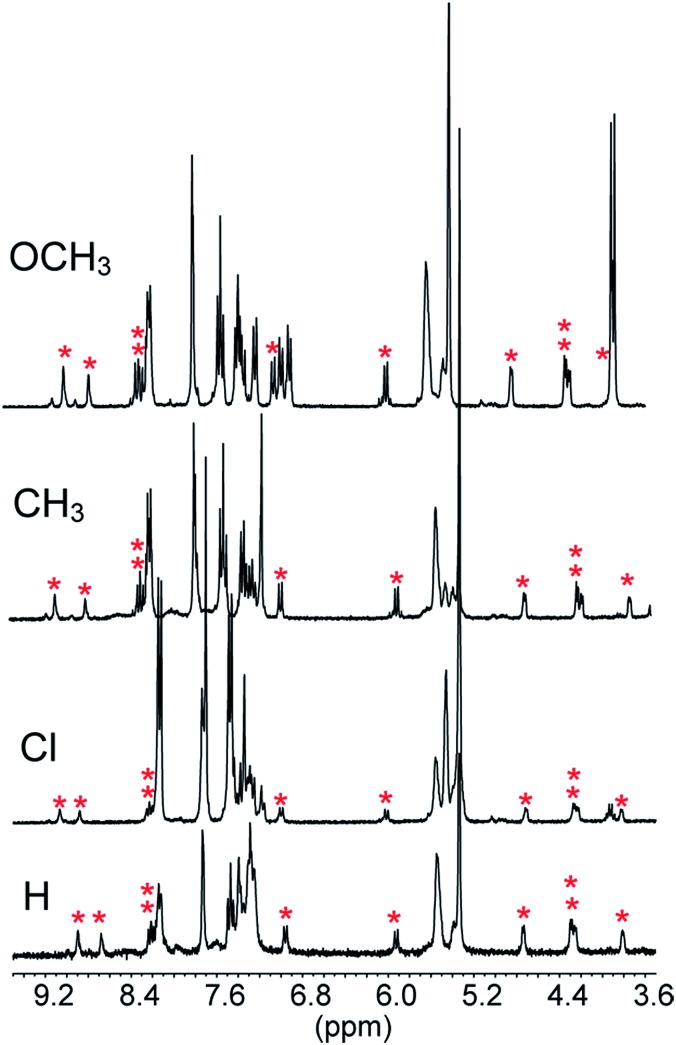
Portions of the ^1^H NMR spectra (CD_2_Cl_2_, 25 °C) recorded in the course of the reaction of 5 mM **1** with equimolar amounts of acid **2** (X = H, Cl, CH_3_ and OCH_3_) after 3.5, 2.5, 7.5 and 44.5 min from the start, respectively. The signals marked with an asterisk are proposed to belong to ten of the twelve protons of the two non-equivalent phenanthroline units in **B′′**. The peak at 5.30 ppm is due to CHDCl_2_.

The reactions of **1** with equimolar amounts of **2** (X = Cl, CH_3_ and OCH_3_) in dichloromethane at 25 °C were monitored by ^1^H NMR spectroscopy (see ESI, Fig. S5, S7 and S8‡). As previously found for the reaction of the parent acid **2** (X = H), the spectra showed that in all cases the sole reaction product was the corresponding decarboxylated compound **3**, besides the catenane in its original neutral state. Furthermore, the ^1^H NMR spectra recorded in the course of the reactions confirmed the presence of transient species, characterized by patterns of signals very similar to each other, as well as to those displayed by the reaction of the parent acid (Fig. 1). Clearly, intermediates of the same nature and with very similar structures (denoted as **B′′** in Scheme 1) are involved in all cases.**
**In our previous work (see ref. 6) the three signals that appear in the range from 4.9 to 3.7 ppm during the reaction of **2** (X = H) (see ESI, Fig. S6‡) were erroneously assigned to the five aromatic protons of the **1**H^+^-bound carbanion. The low precision of the integrated intensities was largely responsible for the incorrect assignment. Whereas the areas of the signals at 4.75 and 3.85 ppm were approximately in a 1 : 1 ratio, that of the signal at 4.30 ppm turned out to be approximately 2.5 times greater than those of the smallest signals. Literature ^1^H NMR data (see ref. 9) showing the non-equivalence of the aromatic protons of the α-methylbenzyl carbanion prompted us to round up the relative intensity of the central peak to 3. The appearance of signals with the same patterns and almost identical chemical shifts during the reaction of **2** (X = Cl, CH_3_ and OCH_3_) (Fig. 1) led us to reconsider our previous assignment, because the presence of *para*-substituents should have altered their number, shape and multiplicity. Hence we concluded that these signals belong to one of the non-equivalent phenanthroline units, which is the common structural element in **B′′** when X is varied, and correspond to four protons in a 1 : 2 : 1 ratio. These signals are shifted upfield by the ring current of the aryl group of the counteranion. We also concluded that the resonances of the aromatic protons of the carbanions should lie in the range from 8.2 to 7.1 ppm. Quite remarkably, the time required to achieve quantitative transformation to **3** amounts to almost two days for the OCH_3_ derivative and decreases in the order OCH_3_ > CH_3_ > H > Cl, becoming as short as a few minutes for the Cl derivative.

**Fig. 2:**
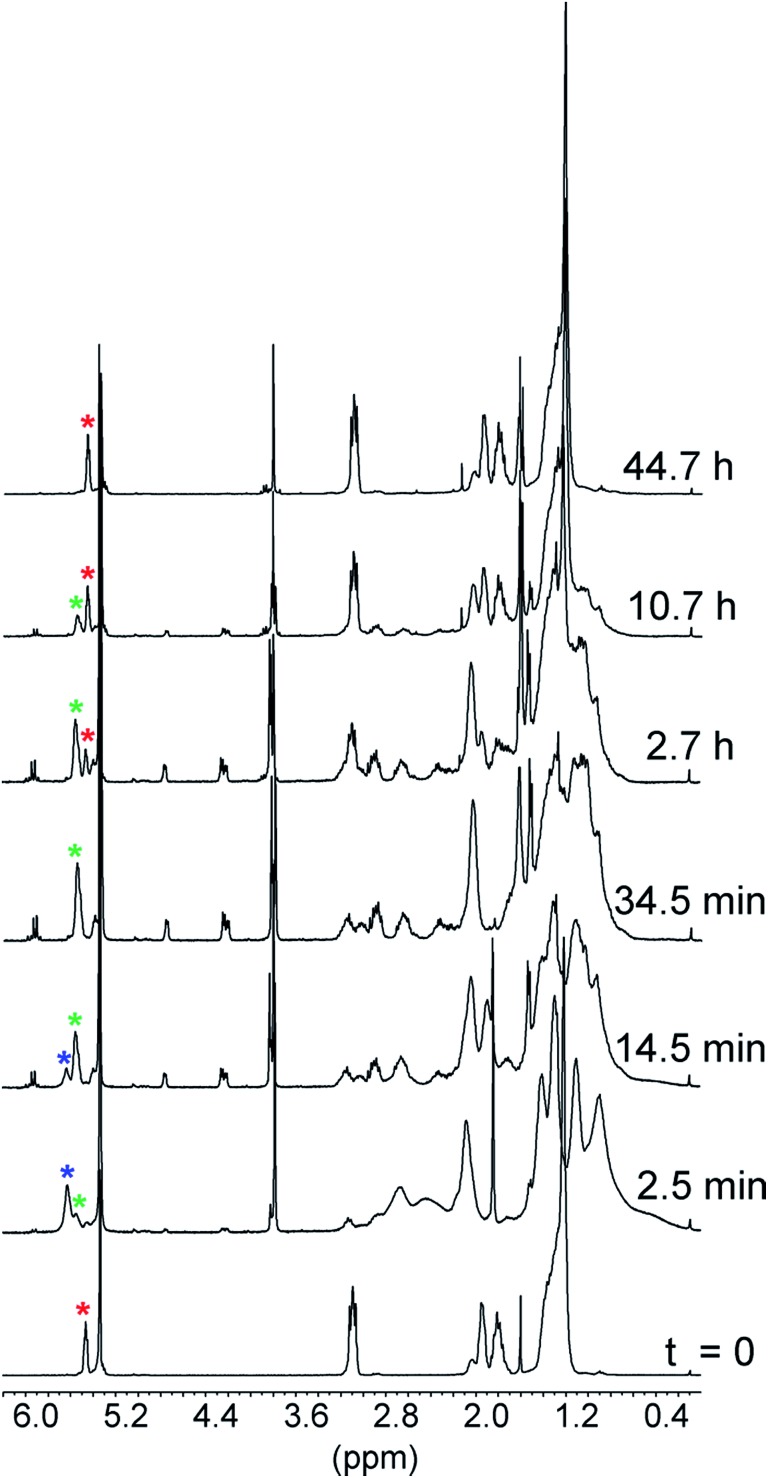
^1^H NMR monitoring of the reaction of 5 mM **1** with 5 mM **2** (X = OCH_3_) in CD_2_Cl_2_ at 25 °C. The trace at time zero is the spectrum of catenand **1**. The asterisks denote the signals of the double bond protons: red = catenand **1**, blue = **B′** and green = **B′′**.

The spectra related to the early stages of the sluggish reaction of **2** (X = OCH_3_) (see Fig. 2 and ESI, Fig. S8‡), particularly in the region of the double bond protons†
†Analysis of the time-dependent changes of the resonances of the double bond protons of **1** is somewhat complicated by the presence of geometrical isomers in a 7 : 1 ratio (see ref. 12a). The pertinent signals are clearly visible in the ^1^H NMR spectrum of the trifluoroacetate salt (see ESI, Fig. S9‡). In the spectrum of **1** the signal of the less abundant isomer is hidden by the signal of CHDCl_2_. at around 5.5 ppm (Fig. 2), gave information about step **B′** → **B′′** which could not be obtained from the faster reactions of **2** (X = CH_3_, H and Cl). The trace at *t* = 2.5 min shows that **1** is no longer in its original, neutral state, but the signals of the ion pair **B′′** are still barely perceptible. This means that the major component of the reaction mixture is the salt denoted by **B′** in Scheme 1. Since the spectrum, particularly in the region of the aromatic protons, is considerably more complex than the spectrum of the trifluoroacetate salt (see ESI, Fig. S9‡), there must be a close association between **1**H^+^ and its carboxylate counterion. The concentration of this transient species is still significant at *t* = 14.5 min and becomes negligibly small in the next spectrum at 34.5 min.

**Fig. 3:**
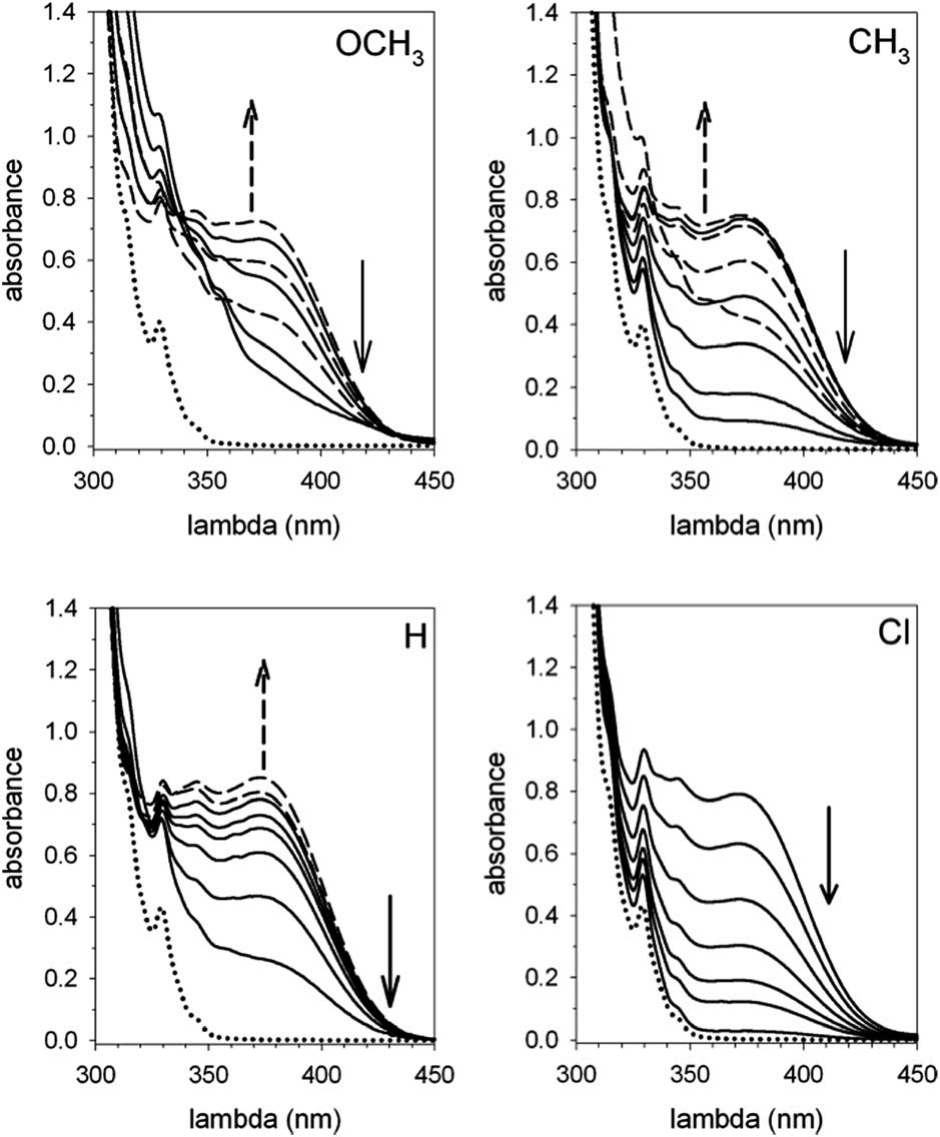
Time dependent UV-vis spectra of the reaction mixtures composed of equimolar amounts (0.30 mM) of catenane **1** and acid **2** (X = OCH_3_, CH_3_, H and Cl) in CH_2_Cl_2_ at 25 °C. The dotted lines represent the absorption spectrum of **1**. The spectra pictured as broken lines refer to the ascending phase of the absorbance, whereas the full lines refer to the descending phase.

**Fig. 4:**
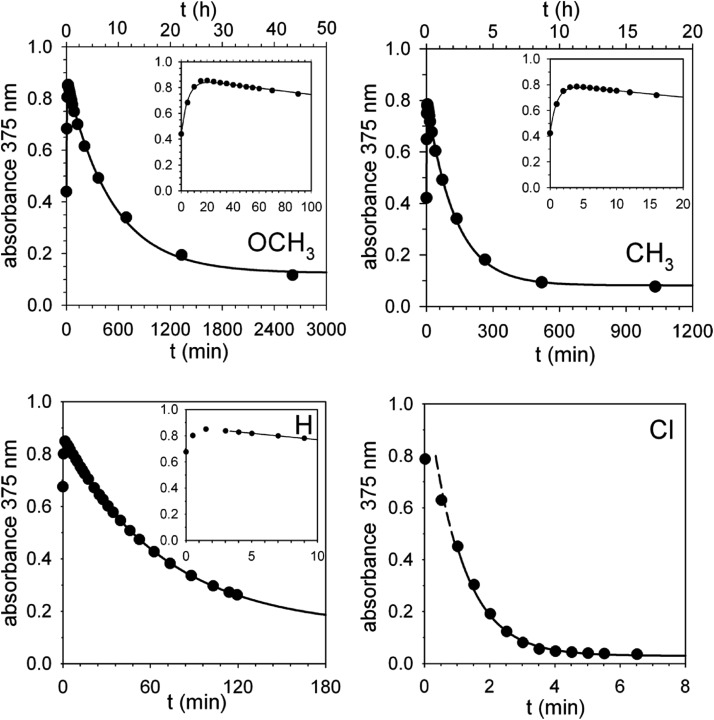
Absorbance *vs.* time profiles of the decarboxylation of 0.30 mM **2** (X = OCH_3_, CH_3_, H and Cl) promoted by 0.30 mM **1** in CH_2_Cl_2_ at 25 °C. X = OCH_3_: the full line is a plot of eqn (S2)‡ with best fit parameters *k*′ = 3.1 × 10^–3^ s^–1^ and *k*′′ = 3.2 × 10^–5^ s^–1^. X = CH_3_: the full line is a plot of eqn (S2)‡ with best fit parameters *k*′ = 1.7 × 10^–2^ s^–1^ and *k*′′ = 1.3 × 10^–4^ s^–1^. X = H: the full line is a plot of a first order equation with *k*′′ = 2.4 × 10^–4^ s^–1^. X = Cl: the full line is a plot of a first order equation with *k*′′ = 2.6 × 10^–2^ s^–1^.

Corroborating evidence came from investigation of the time-dependent UV-vis spectra of the reaction mixtures, which was prompted by the observation that the solutions became yellow in the course of the reactions, but were colourless at the end. The four sets of time-dependent UV-vis spectra (Fig. 3) are characterized by very similar absorption bands (*λ*_max_ = 375 nm) belonging to transient species. The latter were identified with intermediates **B′′** or, more precisely, with their carbanion components, since the absorbance of 0.30 mM **1**H^+^ is 0.30 at 350 nm, drops to 0.05 at 375 nm and becomes negligibly small at 380 nm and beyond. Indeed, the spectra related to the reaction of **2** (X = OCH_3_) (Fig. 4) show that the absorbance reaches its maximum value after about 20 min and then decreases slowly. Clearly, this behavior agrees nicely with the behavior of intermediate **B′′** as monitored by ^1^H NMR spectroscopy (Fig. 2). In the reactions of acids **2** (X = CH_3_ and H) the maximum is reached in about 3 and 1.5 min, respectively, which means that the build-up of **B′′** was complete, or very nearly so, when the first ^1^H NMR spectrum in Fig. S7 and S6,‡ respectively, was recorded. Only the fast decay of the absorption band is visible in the reaction of the Cl derivative, showing that the growing phase was over in the short time required to record the first spectrum after reactant mixing.

For the reactions of **2** (X = OCH_3_ and CH_3_), the absorbance growth and subsequent decay were treated as two separate first-order reactions with rate constants *k*′ and *k*′′, respectively. Fig. 4 shows that there is a good adherence of experimental data to the lines calculated from eqn (S3) (see ESI‡) and the best fit values of *k*′ and *k*′′.

A set of four kinetic runs in which the initial concentration of **1** and **2** (X = OCH_3_) was varied over the range 0.30–1.50 mM (ESI,‡ page S18) gave *k*′ = 2.5 ± 0.5 × 10^–3^ s^–1^ and *k*′′ = 3.1 ± 0.2 × 10^–5^ s^–1^, showing that *k*′ and *k*′′ are concentration-independent quantities. Not surprisingly, the experimental uncertainty in *k*′ is large because of the limited number of data points in the growth phase. As for the reactions of **2** (X = H and Cl), Fig. 4 shows that, except for a brief initial period, there is a strict first-order time dependence of the absorbance data in both cases.

**Fig. 5:**
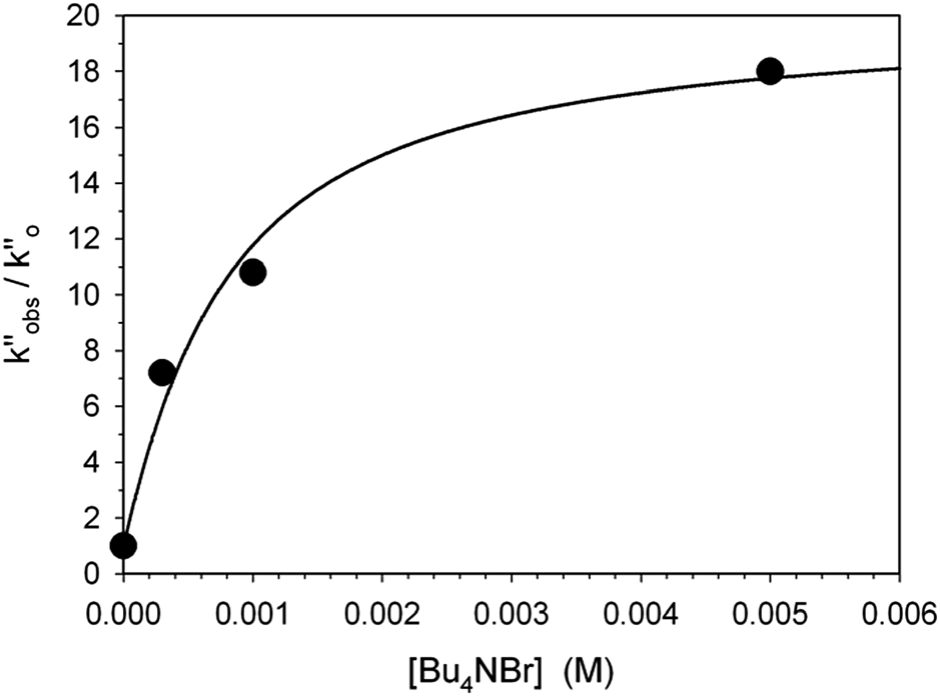
Effect of the added Bu_4_NBr on the rate of decarboxylation of 0.30 mM **2** (X = H) promoted by 0.30 mM **1** in CH_2_Cl_2_ at 25 °C. The full line is a plot of eqn (1) with best fit parameters *K*_ass_ = 1600 M^–1^ and *k*′′_salt_ = 5.0 × 10^–3^ s^–1^.

**Scheme 3:**
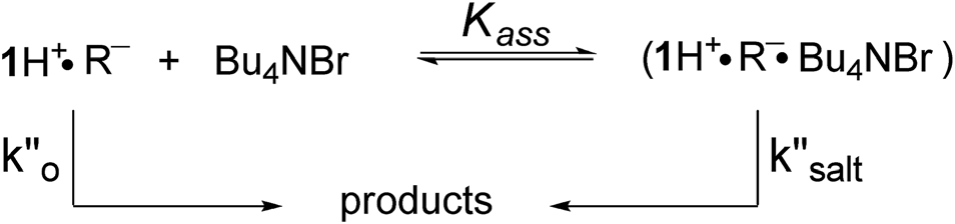
Associative mechanism involving the fast and reversible formation of a reactive 1 : 1 adduct between the added salt and the ion pair **1**H^+^R^–^. The latter corresponds to **B′′** in Scheme 1.

In a last set of rate measurements the effect of added Bu_4_NBr on the rate of reaction of 0.30 mM **2** (X = H) with equimolar **1** was investigated. UV-vis monitoring of the reactions showed in all cases a rapid growth followed by a slower decay of the absorption band centered at 375 nm, the shape and maximum intensity of which were hardly affected by the added salt, even at the highest salt concentration of 5.0 mM (Fig. S10‡). Again, except for a brief initial period, the absorbance was found to decrease according to first-order kinetics. The first order rate constants (*k*′′_obs_) increased with increasing salt concentration and showed a marked tendency to saturate. Fig. 5 shows a close adherence of the kinetic data to the line of eqn (1), which is consistent with the reaction mechanism depicted in Scheme 3, where the salt forms a 1 : 1 complex of moderate stability with **B′′** (*K*_ass_ = 1600 M^–1^), which is significantly more reactive than uncomplexed **B′′** (*k*′′_salt_/*k*′′_o_ = 20).‡
‡An alternative mechanism involves fast and reversible double exchange between ion pair partners, followed by a rate limiting second-order reaction between **1**H^+^Br^–^ and Bu_4_N^+^R^–^.
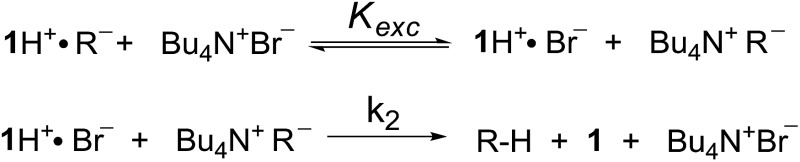
 It should be stressed that this mechanism, which is almost kinetically indistinguishable from that in Scheme 3 (see ESI,‡ page S20), also proceeds through an ion–quartet transition state..

It seems likely that the components of the ion pair **B′′**, namely **1**H^+^ and the carbanion derived from **2** (X = H), are brought into an orientation more adapted to proton transfer when **B′′** becomes a component of an ion quartet as a result of association with Bu_4_NBr. However, due to the lack of structural information on the ion quartet, a detailed interpretation of the rate enhancing effect exerted by the added salt does not appear to be accessible.
1

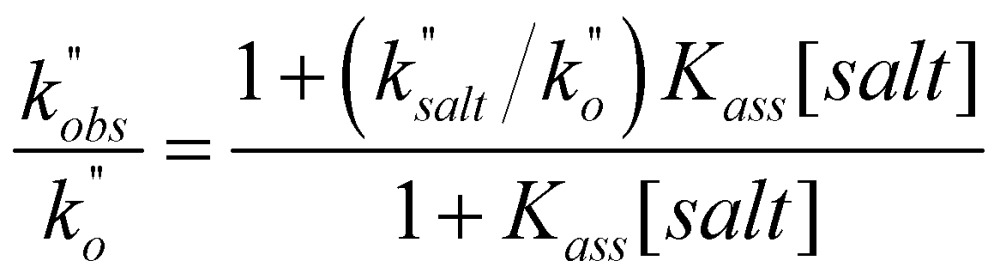




To sum up, the combination of data from ^1^H NMR and UV-vis spectroscopy strongly supports the operation of the reaction mechanism outlined in Scheme 1, which involves compounds **B′** and **B′′** as reaction intermediates.

Data from UV-vis spectroscopy gave pieces of information which could not be obtained from ^1^H NMR spectroscopy. Firstly, the transient absorption band at 375 nm provided direct evidence of the existence and kinetic behavior of the carbanion intermediates, whose signals in the ^1^H NMR spectra were hardly visible. Secondly, the kinetics based on UV-vis spectroscopy gave qualitative information about the substituent effects on the rate of step **B′** → **B′′**. The reactivity order Cl > H > CH_3_ > OCH_3_ is the same as that observed in the reactions promoted by Et_3_N, but the rupture of the R–CO_2_^–^ bond is much faster in the reactions promoted by **1** because any stabilization of R–CO_2_^–^ by hydrogen bonding is hardly conceivable when **1**H^+^ is the countercation.§

**Fig. 6:**
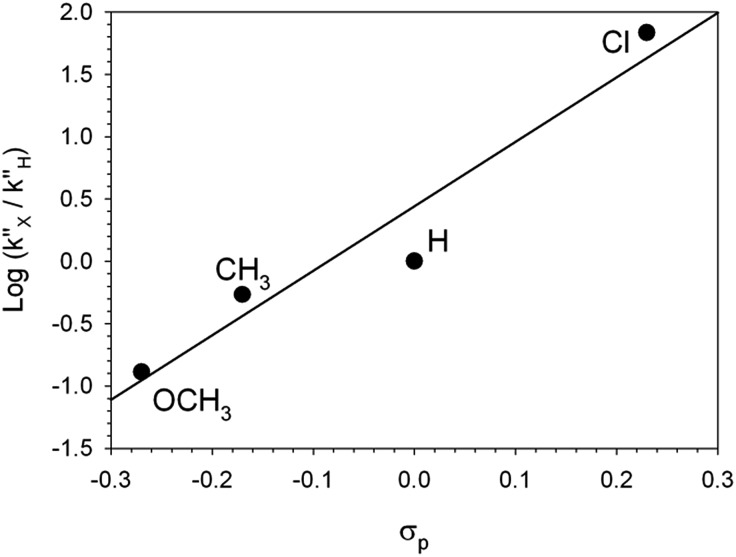
Hammett plot for the decarboxylation of 2-cyano-2-arylpropionic acids **2**. The *k*′′ values are from the caption of Fig. 4.

Finally, kinetic treatment of the UV-vis data provided a quantitative estimate of the effect of the substituents on the rate of the back proton transfer (step **B′′** → **A**), whereas only a qualitative reactivity order could be obtained from the ^1^H NMR spectra. The Hammett plot of log (*k*′′_x_/*k*′′_H_) *vs. σ*_p_ reported in Fig. 6 gives *ρ* = 5.2 (*r* = 0.98). Such a large *ρ* value is indicative of a much larger fraction of negative charge on the benzyl carbon atom in the transition state, which is equivalent to saying that the kinetic basicity of the anionic component in **B′′** is enhanced by EWGs and depressed by EDGs. Unusual as it might seem, such a behavior is not unprecedented.^10^ Bordwell *et al.*10*b* carried out an extensive investigation of the equilibrium and kinetic acidities of ArCHMeNO_2_ (12 substituents). They found that the rates of protonation of ArCMe=NO_2_^–^ were enhanced by EWGs and retarded by EDGs (*ρ* > 0) and concluded that there is a very little delocalization of the fractional negative charge to the oxygen atoms in the transition state, whereas the negative charge is mostly localized on the oxygen atoms of the nitronate anions.

**Fig. 7:**
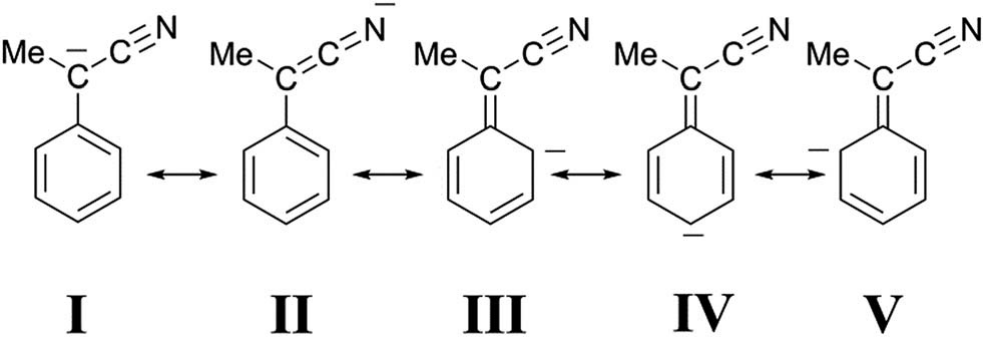
Canonical structures of 1-cyano-1-phenyl ethanide.

Although a cyano group is less effective than a nitro group in stabilizing a negative charge on an adjacent carbon atom, we suggest that a similar interpretation applies to the protonation of our cyano-stabilized carbanions. Accordingly, it seems likely that the canonical structure **I** (Fig. 7), unlike structure **II**, contributes very little to the hybrid. This view is strongly supported by the X-ray molecular structure of the Bu_4_N^+^ salt of 1-cyano-1-phenylethanide, prepared by the treatment of **2** (X = H) with Bu_4_NOH.^11^ The essential planarity of the anion, coupled with the finding that only the nitrogen of the cyano group is involved in multiple hydrogen bonding interactions with the α-methylene groups of the cations, is indicative of very little delocalization of the negative charge to the sp^2^ benzylic carbon atom, as well as significant delocalization to the nitrogen atom of the cyano group. In our case, a high negative charge density on the nitrogen atom of the CN group would guarantee a strong electrostatic stabilization of **B′′**, whose shape is supposed to be one in which the group =C=N^–^ points to the proton embedded in the two-phenanthroline core.

## Experimental section

### Instruments and methods

^1^H NMR spectra were recorded on a 300 MHz spectrometer. The spectra were internally referenced to the residual proton signal of the solvent at 5.30 ppm. Spectrophotometric measurements were carried out on a diode array spectrophotometer equipped with a thermostated cell compartment. Mass spectrometric measurements were carried out using an ESI-TOF spectrometer.

### Materials

All reagents and solvents were purchased at the highest commercial quality and were used without further purification unless otherwise stated. CH_2_Cl_2_ and CD_2_Cl_2_ were flushed through basic alumina immediately prior to use. The same batch of solvent was used in each of the sets of kinetic measurements. Et_3_N was distilled from metallic sodium before use. Catenane **1** (a mixture of the EE, EZ and ZZ geometrical isomers) was available from a previous investigation.^12^ Acid **2** (X = H) was available from a previous investigation^6^ while acids **2** (X = Cl, CH_3_ and OCH_3_) were prepared from the corresponding ethyl esters^13^ according to a literature procedure.^14^

#### 2-Cyano-2-(4′-chlorophenyl)propionic acid (**2**, X = Cl)

This compound had a m.p. of 81–82 °C, lit. 81–82 °C.^15^^1^H NMR (300 MHz, CD_2_Cl_2_) *δ*: 7.55–7.52 (m, 2H), 7.46–7.43 (m, 2H), 3.52 (br s, 1H), 1.99 (s, 3H).

#### 2-Cyano-2-(4′-methyl)propionic acid (**2**, X = CH_3_)

This new compound had a m.p. of 86–88 °C (dec.). ^1^H NMR (300 MHz, CDCl_3_) *δ*: 7.46–7.43 (m, 2H), 7.24–7.22 (m, 2H), 4.05 (br. s, 1H), 2.36 (s, 3H), 1.97 (s, 3H). ^13^C NMR (75 MHz, CDCl_3_) *δ*: 171.2, 139.1, 131.8, 129.8, 125.7, 119.0, 47.7, 24.2, 20.9. UV-vis (CH_2_Cl_2_): *λ*_max_ (*ε*) = 230 nm (3200 cm^–1^ M^–1^), 262 nm (400 cm^–1^ M^–1^), 285 nm (200 cm^–1^ M^–1^). ESI-MS (negative-ion-mode): 144 (M – H^+^ – CO_2_).

#### 2-Cyano-2-(4′-methoxyphenyl)propionic acid (**2**, X = OCH_3_)

This new compound had a m.p. of 87–89 °C (dec.). ^1^H NMR (300 MHz, CD_2_Cl_2_) *δ*: 7.51–7.46 (m, 2H), 6.99–6.93 (m, 2H), 4.95 (br. s, 1H), 3.82 (s, 3H), 1.97 (s, 3H). ^13^C NMR (75 MHz, CD_2_Cl_2_) *δ*: 171.3, 160.0, 127.1, 126.7, 119.2, 114.3, 55.3, 47.1, 23.8. UV-vis (MeOH): *λ*_max_ (*ε*) = 230 nm (13 900 cm^–1^ M^–1^), 274 nm (1600 cm^–1^ M^–1^), 281 nm (1300 cm^–1^ M^–1^). ESI-MS (negative-ion-mode): 160 (M – H^+^ – CO_2_).

## Conclusions

In this work we have described the first example of a chemically operated molecular switch in which the rate of the back and forth motion could be regulated within wide limits by variations in the fuel structure, namely by variations in the nature of the *para*-substituent of the 2-cyano-2-arylpropanoic acid used as a fuel. The time taken to complete a full cycle decreased in the order OCH_3_ > CH_3_ > H > Cl. It was as long as two days for the OCH_3_ derivative, and dropped to a few minutes for the Cl derivative. Fine tuning of the motion rate could be further achieved by the addition of Bu_4_NBr. A detailed kinetic investigation has shown the intermediacy of ion pairs **B′** and **B′′**, which differ in the nature of the anionic component. The negative charge of the anion in **B′′** was suggested to be mostly localized on the nitrogen atom of the CN group, and strongly shifted to the benzyl carbon atom in the transition state of the rate-determining proton transfer step.

## Conflicts of interest

There are no conflicts to declare.

## Supplementary Material

Supplementary information
