# Supplementary material for: Variations in the fuel structure control the rate of the back and forth motions of a chemically fuelled molecular switch
Source: Chem Sci. 2017 Oct 18;9(1):181–8. doi: 10.1039/c7sc04123c (PMC5869305; doi:10.1039/c7sc04123c)
Supplement: Supplementary file 1 [file SC-009-C7SC04123C-s001.pdf]

Electronic Supplementary Material (ESI) for Chemical Science.
This journal is © The Royal Society of Chemistry 2017

Electronic Supplementary Information for

Variations in the Fuel Structure Control the Rate of the Back and Forth Motions of a Chemically Fuelled Molecular Switch

*Chiara Biagini,[a] Simone Albano,[a] Rachele Caruso,[a] Luigi Mandolini,[a] José Augusto Berrocal[b] and Stefano Di Stefano[a]**

[a]*Dipartimento di Chimica and Istituto CNR di Metodologie Chimiche-IMC, Sezione Meccanismi di Reazione c/o Dipartimento di Chimica, Università degli Studi di Roma "La Sapienza", P.le A. Moro 5, 00185 Rome, Italy.*

[b]*Institute for Complex Molecular Systems, Eindhoven University of Technology, P.O. Box 513, 5600 MB Eindhoven (The Netherlands)*

Contents

^{1}H NMR spectrum of acid **2**, X = OCH_3	S3
^{13}C NMR spectrum of acid **2**, X = OCH_3	S4
^{1}H NMR spectrum of acid **2**, X = CH_3	S5
^{13}C NMR spectrum of acid **2**, X = CH_3	S6
^{1}H NMR spectrum of acid **2**, X = Cl	S7
^{1}H NMR monitoring of the reaction between **2**, X = Cl and Et_3N	S8
^{1}H NMR monitoring of the reaction between **2**, X = OCH_3 and Et_3N	S9
^{1}H NMR monitoring of the reaction between **2**, X = CH_3 and Et_3N	S10
Hammett plot for reaction between **2**, X = OCH_3, CH_3, H and Cl with Et_3N	S11
^{1}H NMR monitoring of the reaction between **1** and **2**, X = Cl	S12
^{1}H NMR monitoring of the reaction between **1** and **2**, X = H	S13
^{1}H NMR monitoring of the reaction between **1** and **2**, X = CH_3	S14
^{1}H NMR monitoring of the reaction between **1** and **2**, X = OCH_3	S15
^{1}H NMR spectrum of **1**H^+ $CF_3CO_2^-$ in CD_2Cl_2	S16
Consecutive first-order reactions	S17
Effect of reactant concentration	S18
Comparison of the UV-Vis spectra of **B”** in the presence and absence of added salt	S19
Alternative mechanisms for reaction of **2**, X = H and **1** in the presence of Bu_4NBr	S20

^{1}H NMR spectrum acid **2**, X = OCH_3

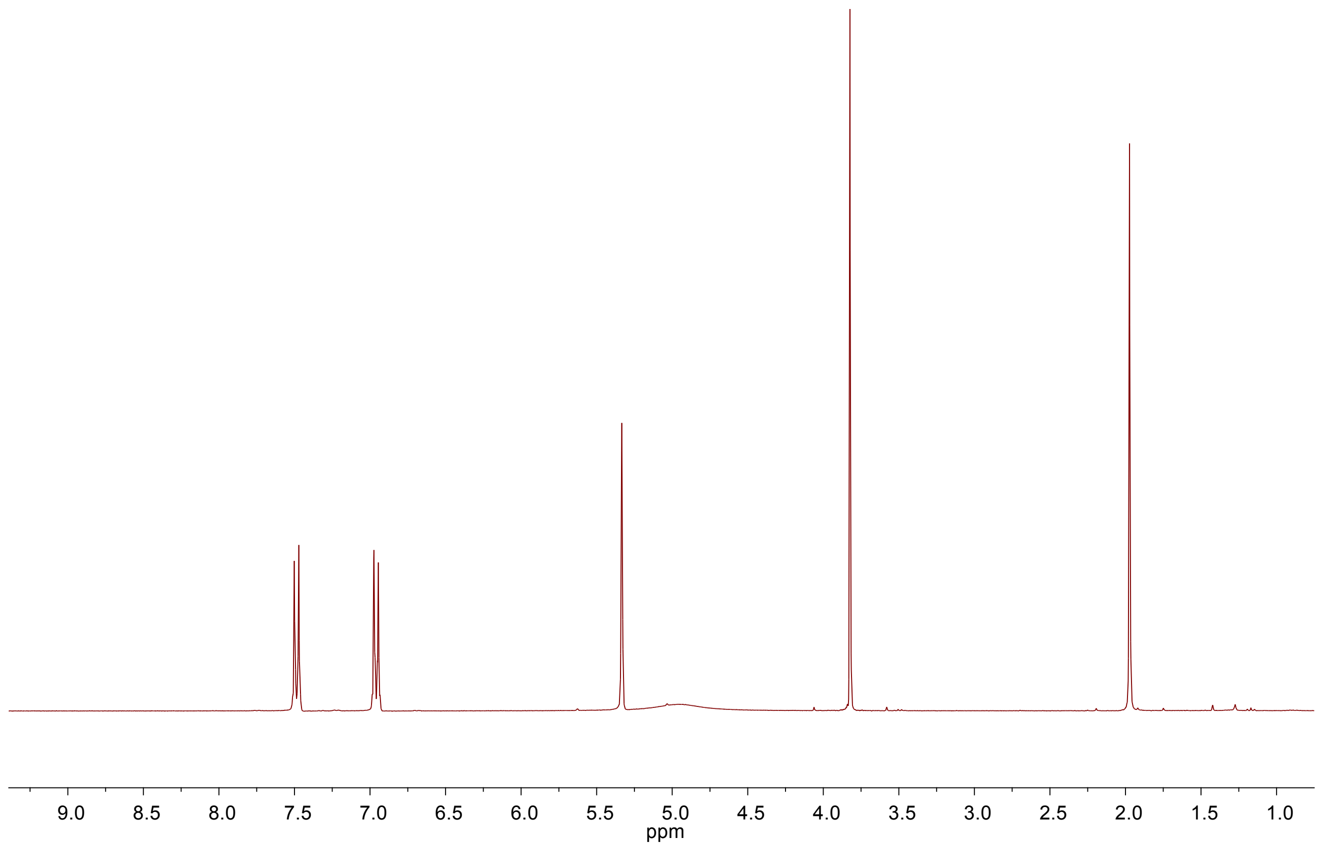

^{1}H NMR (300 MHz, CD_2Cl_2)

δ: 7.51-7.46 (m, 2H), 6.99-6.93 (m, 2H), 4.95 (br. s, 1H), 3.82 (s, 3H), 1.97 (s, 3H).

^{13}C NMR spectrum of acid **2**, X = OCH_3

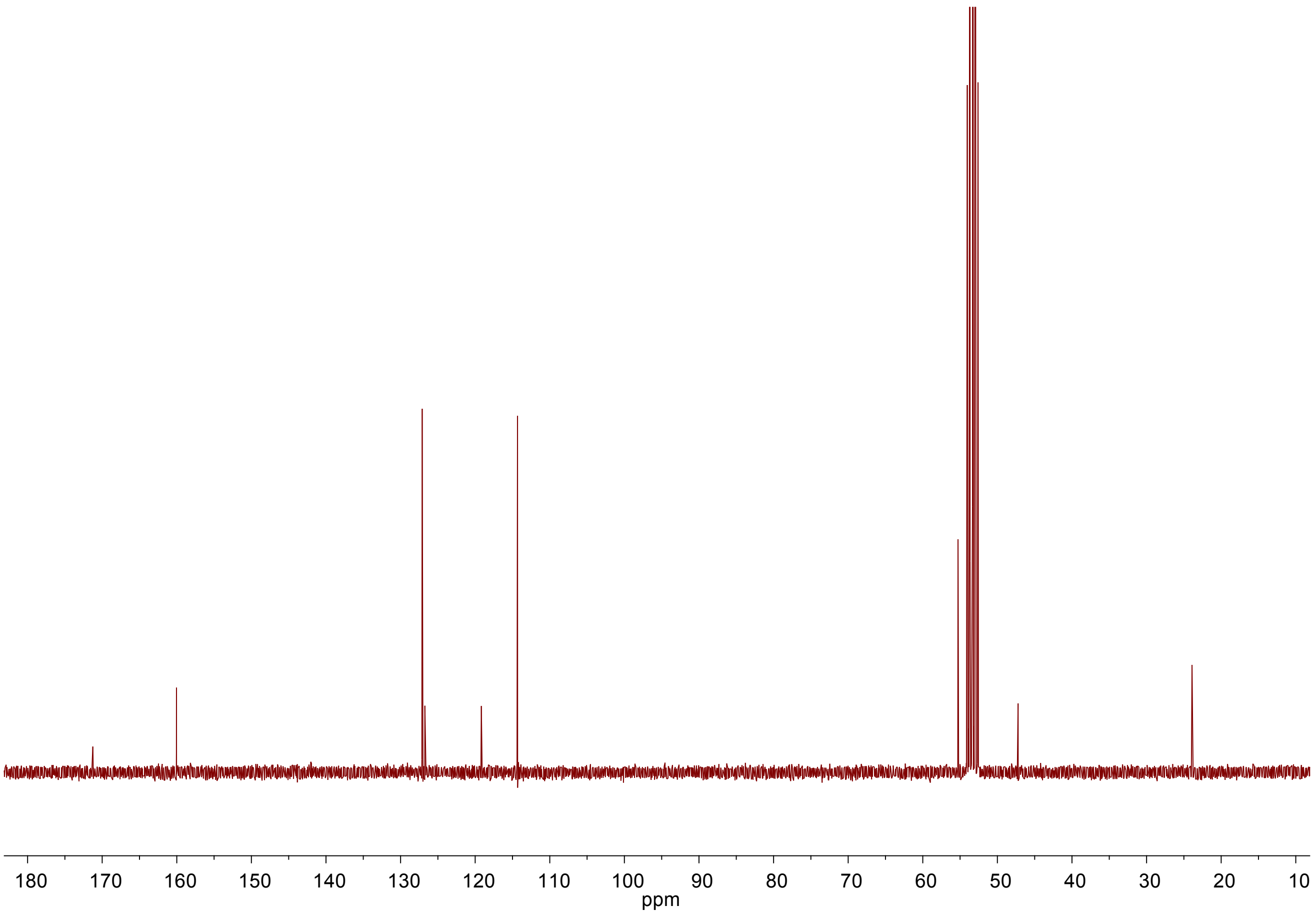

^{13}C NMR (75 MHz, CD_2Cl_2)

δ: 171.3, 160.0, 127.1, 126.7, 119.2, 114.3, 55.3, 47.1, 23.8.

^{1}H NMR spectrum acid **2**, X = CH_3

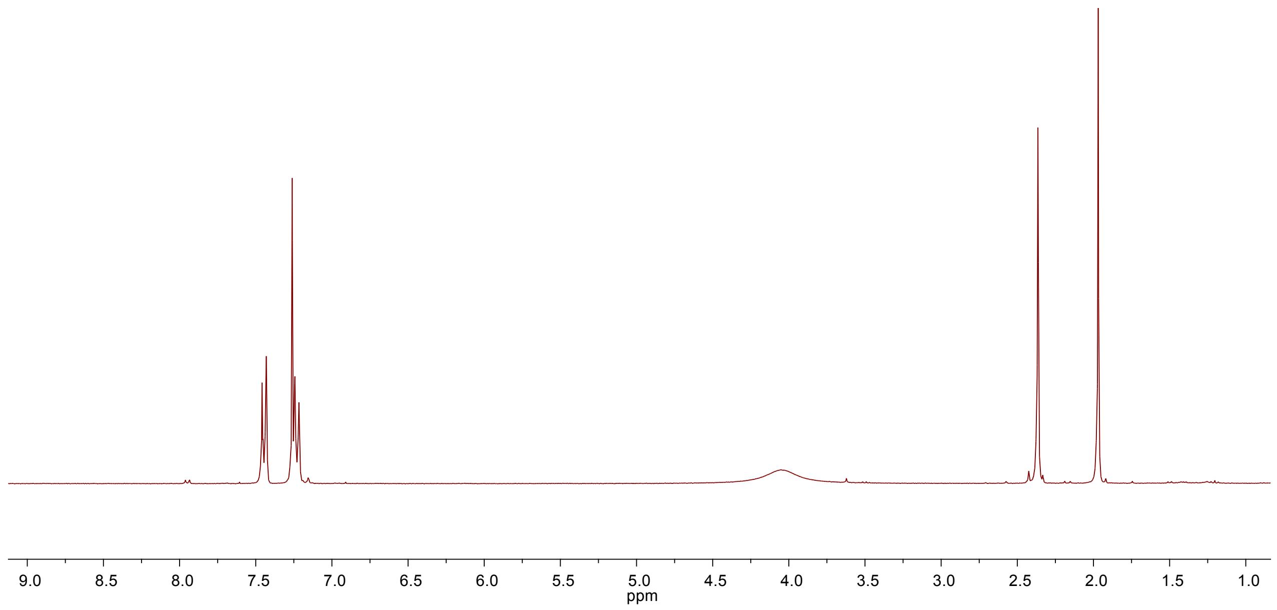

^{1}H NMR (300 MHz, $CDCl_3$)

δ: 7.46-7.43 (m, 2H), 7.24-7.22 (m, 2H), 4.05 (br. s, 1H), 2.36 (s, 3H), 1.97 (s, 3H).

^{13}C NMR spectrum of acid **2**, X = CH_3

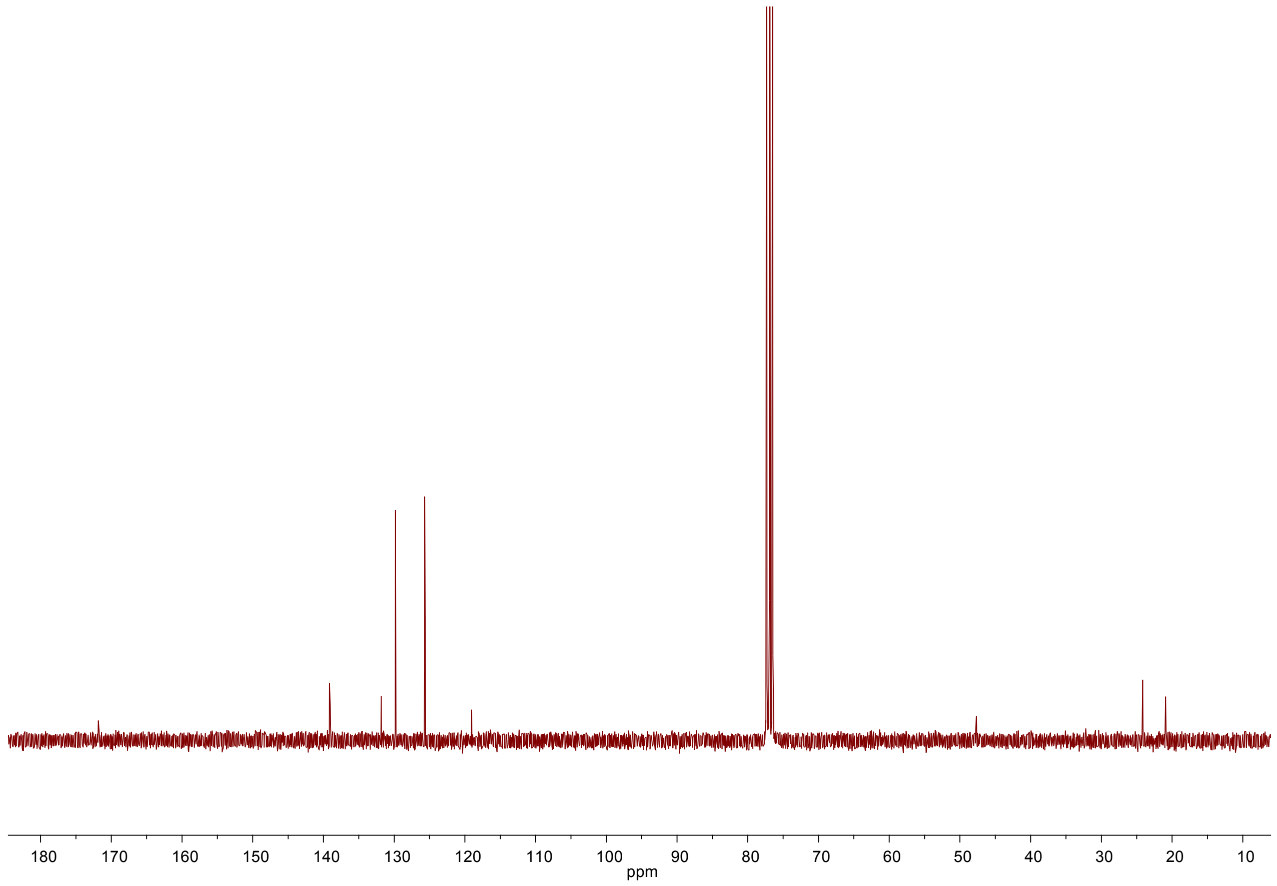

^{13}C NMR (75 MHz, $CDCl_3$)

δ: 171.2, 139.1, 131.8, 129.8, 125.7, 119.0, 47.7, 24.2, 20.9.

^{1}H NMR spectrum acid **2**, X = Cl

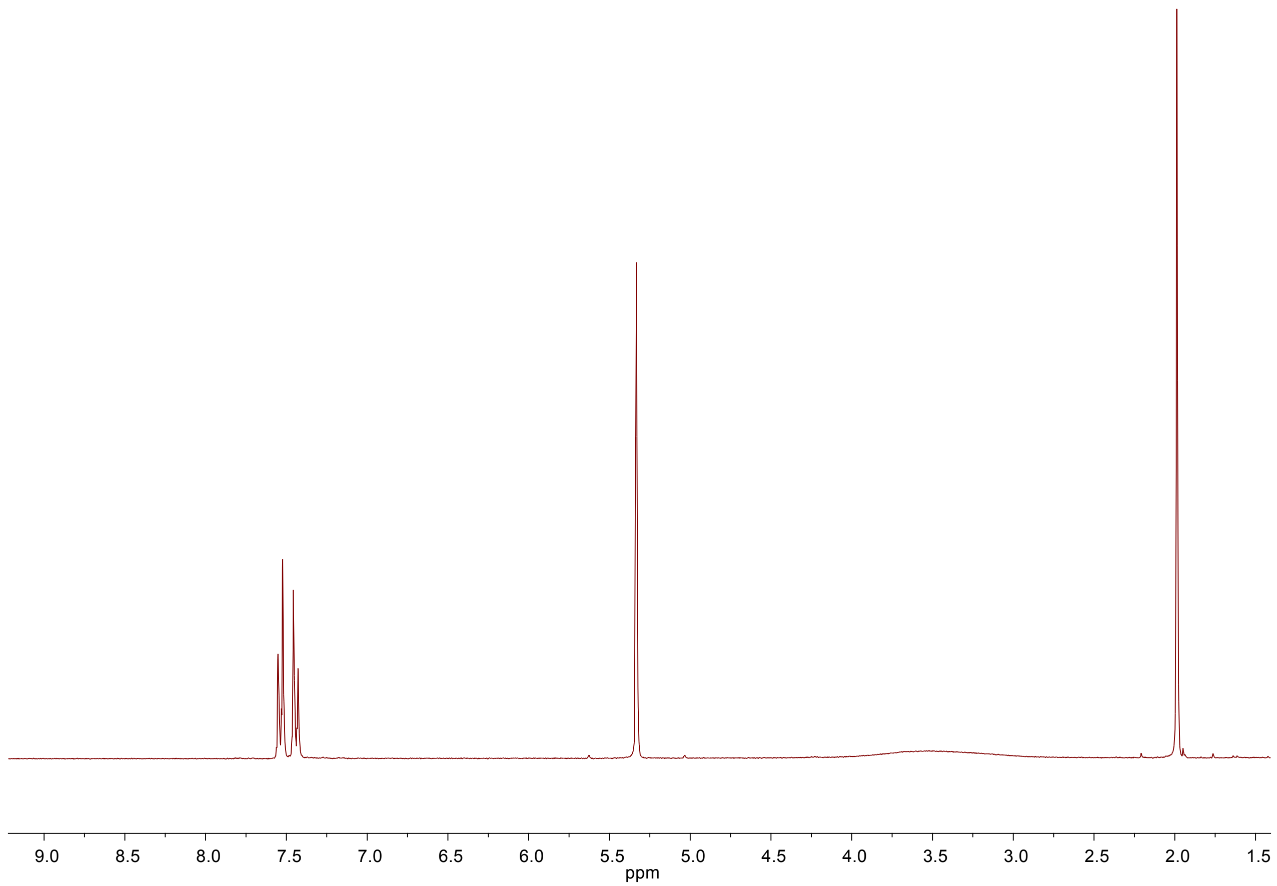

^{1}H NMR (300 MHz, CD_2Cl_2)

δ: 7.55–7.52 (m, 2H), 7.46-7.43 (m, 2H), 3.52 (br s, 1H), 1.99 (s, 3H).

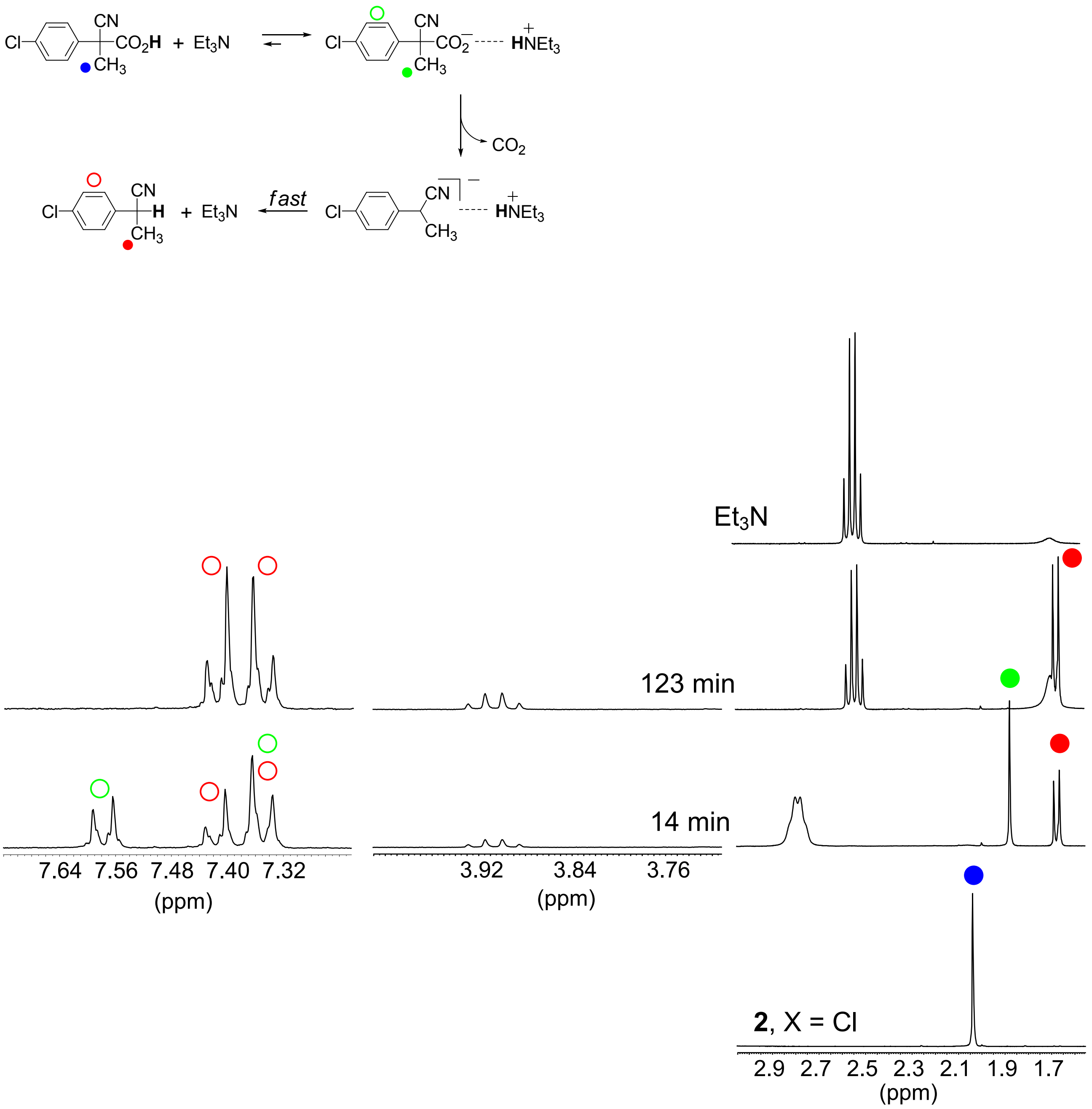

Fig. S1. Decarboxylation of 10 mM **2**, X = Cl promoted by 10 mM Et_3N in CD_2Cl_2 at 25 °C. Portion of ^1H NMR spectra recorded at $t \approx t_{½}$ (14 min) and $t = \infty$ (123 min). Portions of the spectra of the pure reactants are shown for comparison. The broad signal is due to adventitious H_2O.

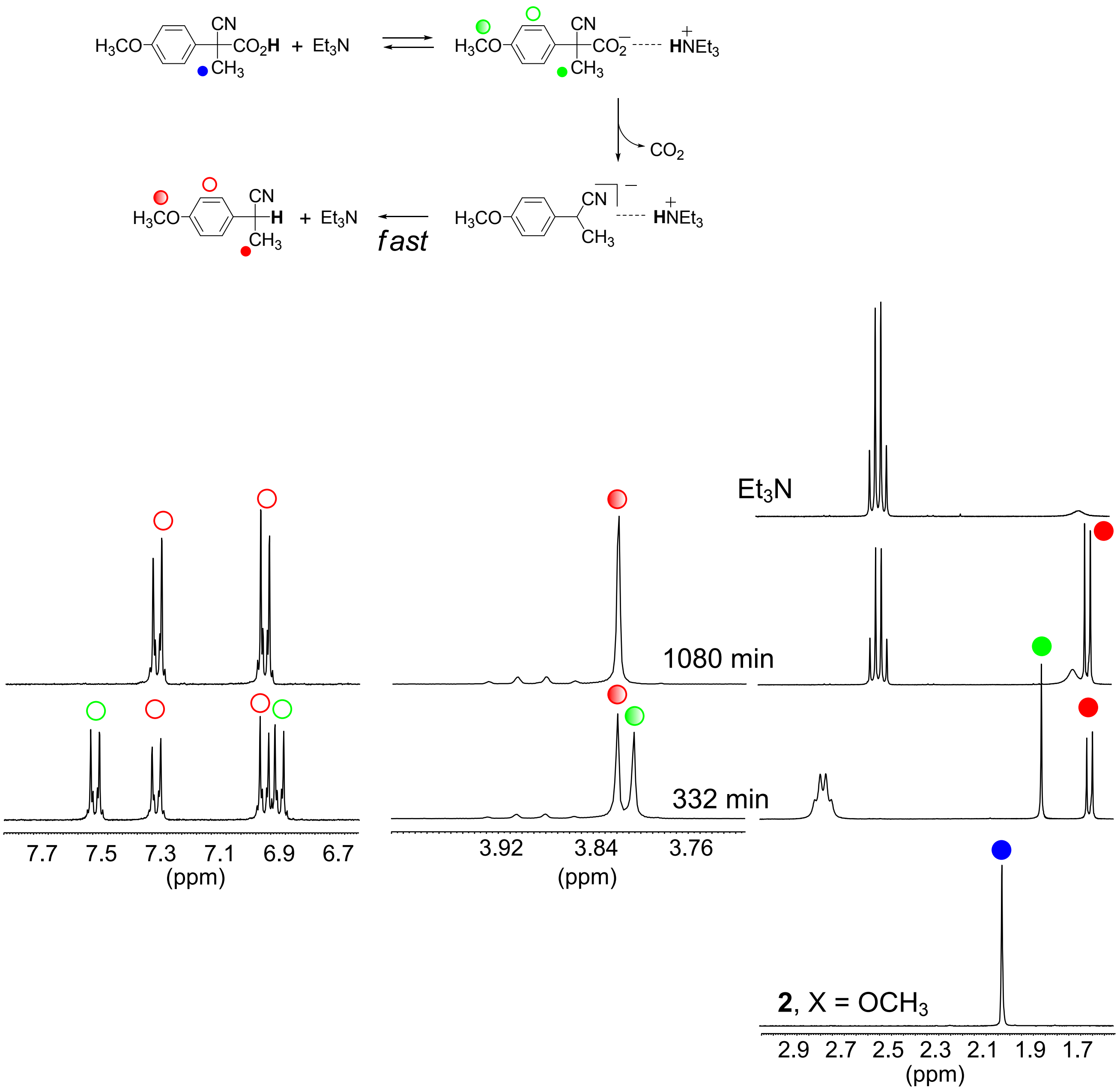

Fig. S2. Decarboxylation of 10 mM **2**, X = OCH_3 promoted by 10 mM Et_3N in CD_2Cl_2 at 25 °C. Portion of ^1H NMR spectra recorded at $t \approx t_{½}$ (332 min) and $t = \infty$ (1080 min). Portions of the spectra of the pure reactants are shown for comparison. The broad signal is due to adventitious H_2O.

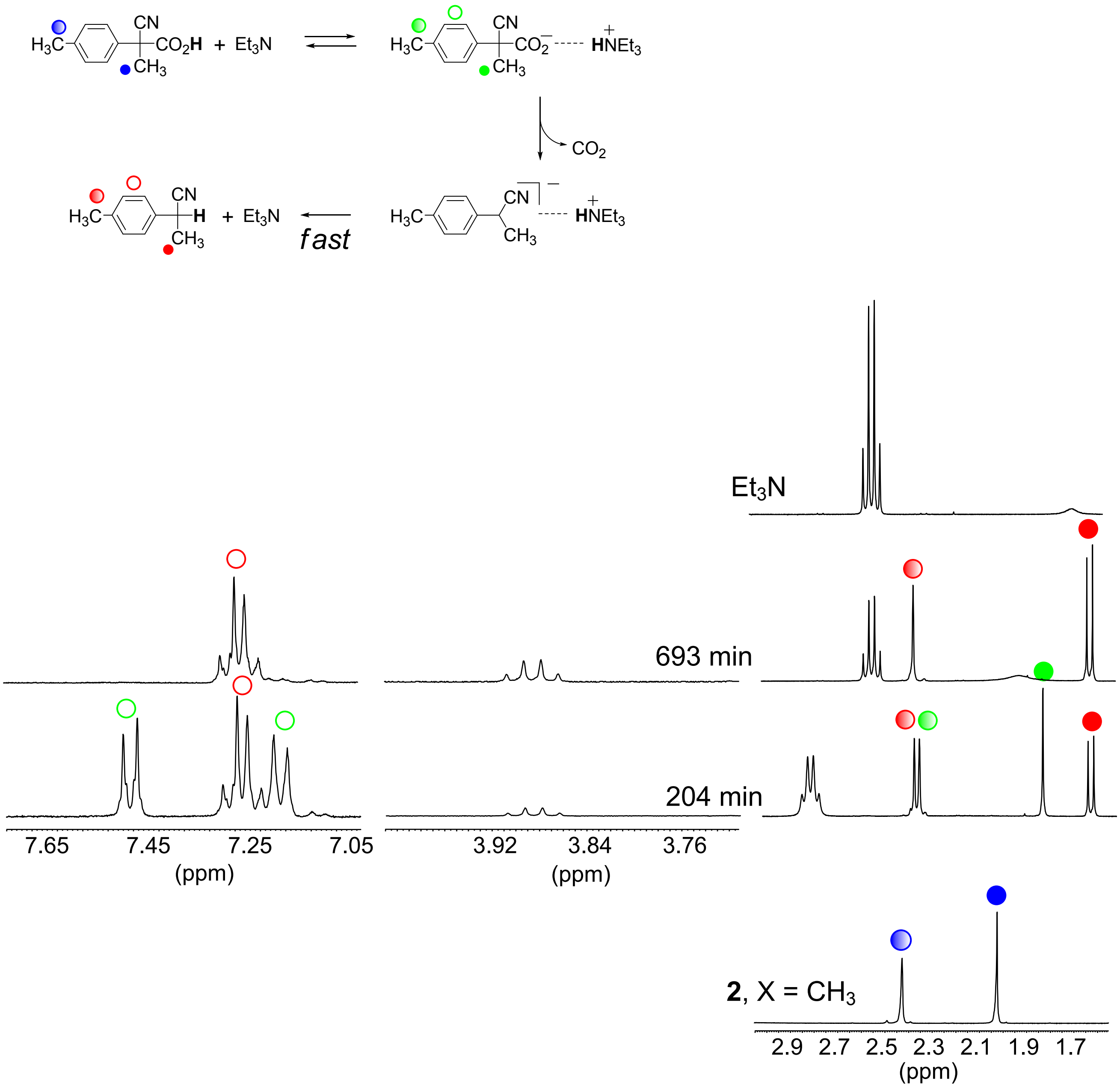

Fig. S3. Decarboxylation of 10 mM **2**, X = CH_3 promoted by 10 mM Et_3N in CD_2Cl_2 at 25 °C. Portion of ^{1}H NMR spectra recorded at t ≈ $t_{½}$ (204 min) and t = ∞ (693 min). Portions of the spectra of the pure reactants are shown for comparison. The broad signal is due to adventitious H_2O.

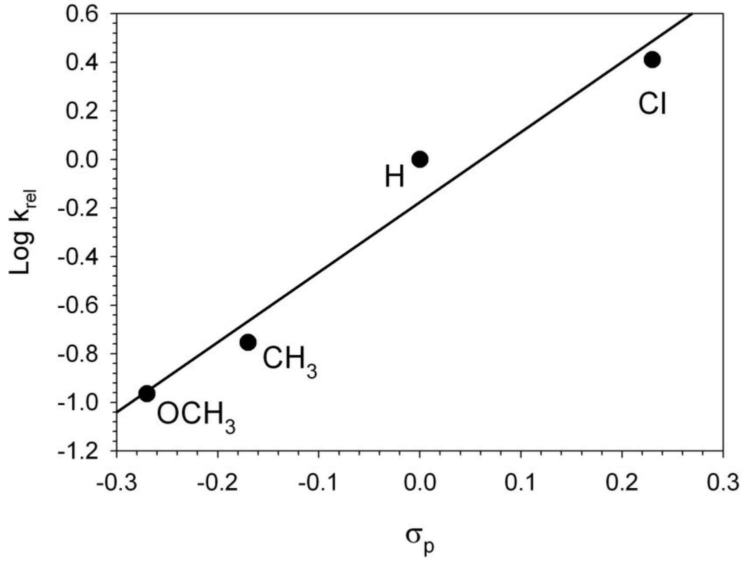

$\rho = 2.9$

$r = 0.9816$

Fig. S4. Hammett plot related to reactions between 10 mM **2**, X = OCH_3, CH_3 , H, and Cl with 10 mM Et_3N in CD_2Cl_2 at 25 °C.

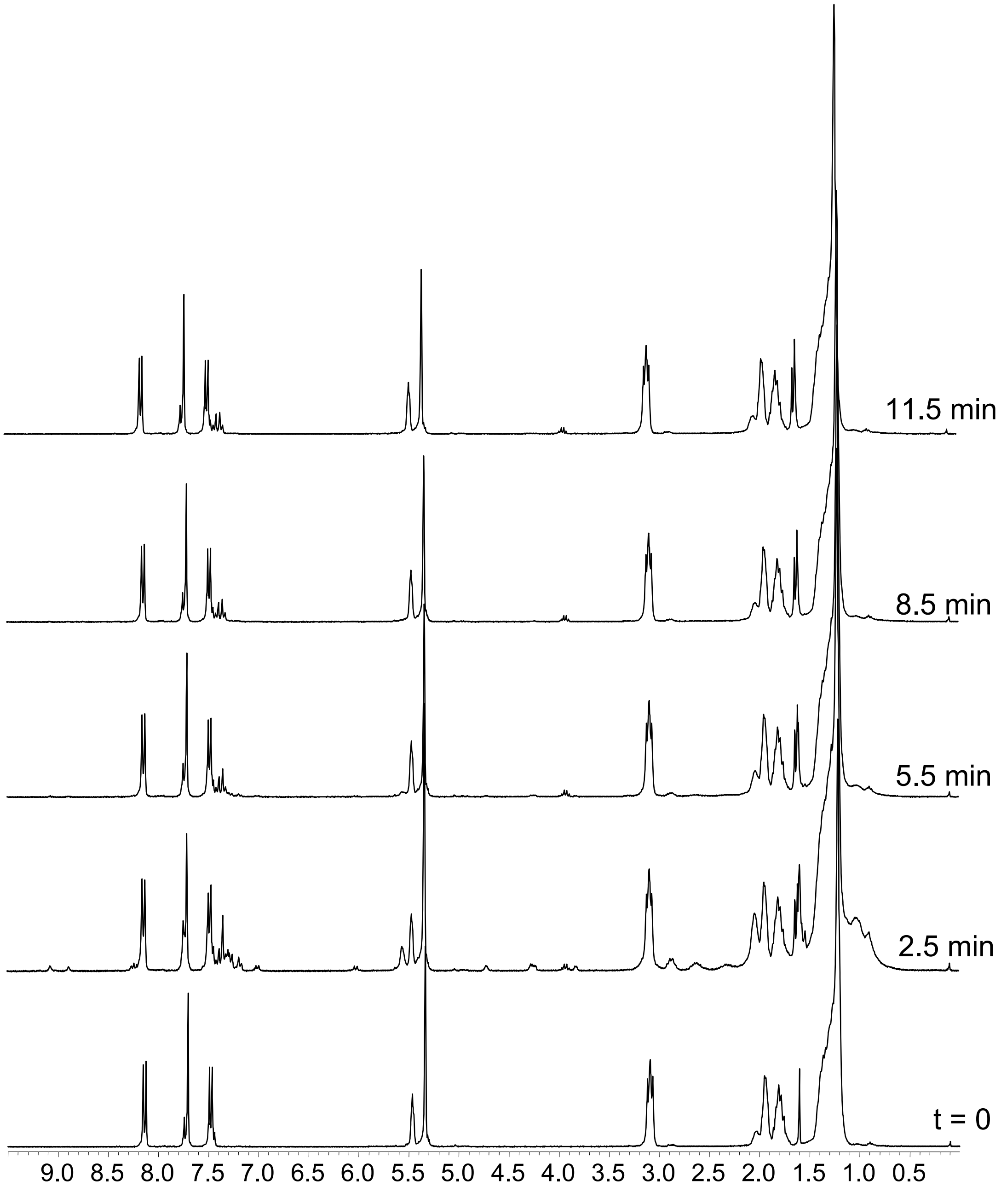

Fig. S5. ^{1}H NMR monitoring of the reaction between 5 mM **1** and 5 mM **2**, X = Cl in CD_2Cl_2 at 25 °C.

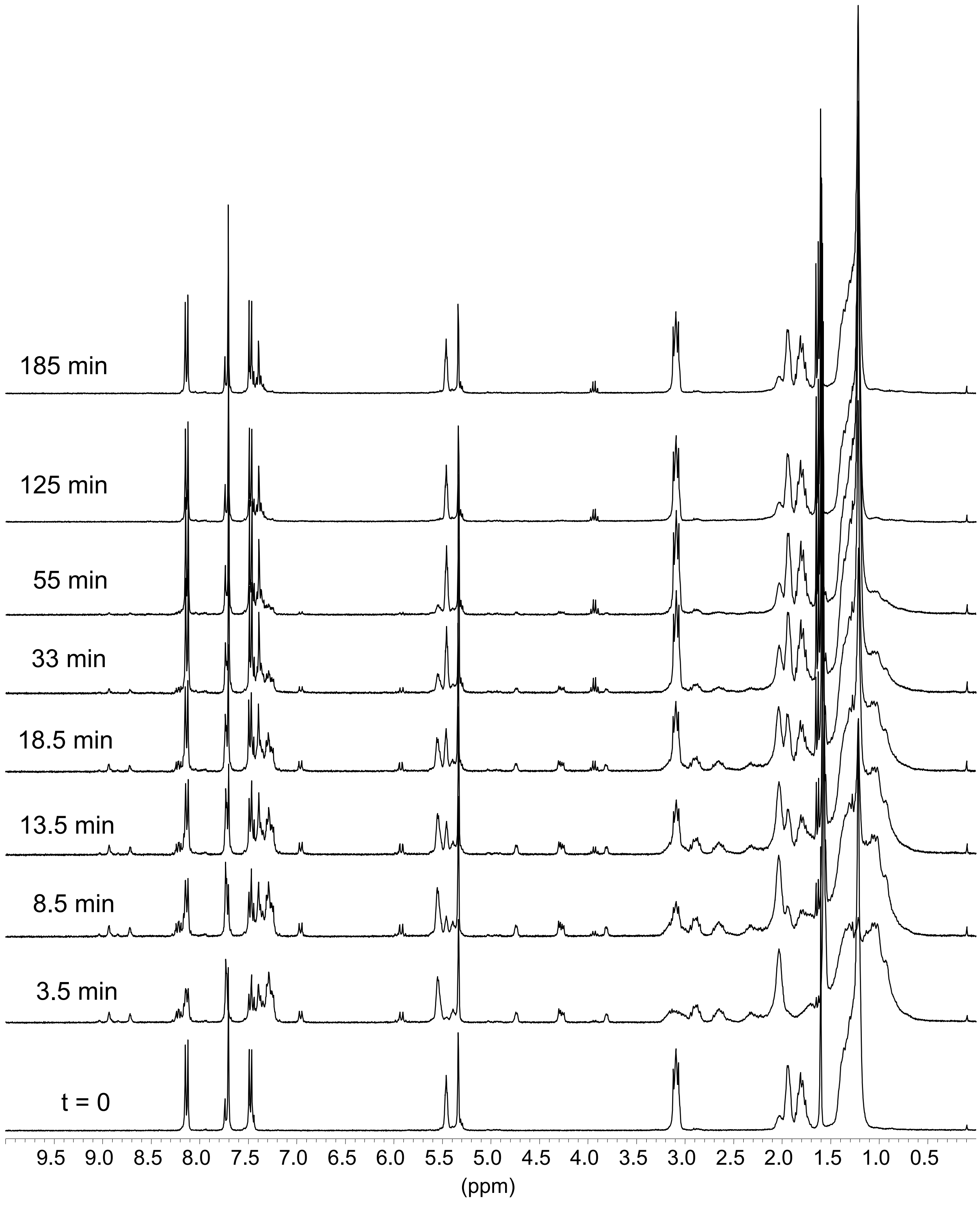

Fig. S6. ^{1}H NMR monitoring of the reaction between 5 mM **1** and 5 mM **2**, X = H in CD_2Cl_2 at 25 °C.

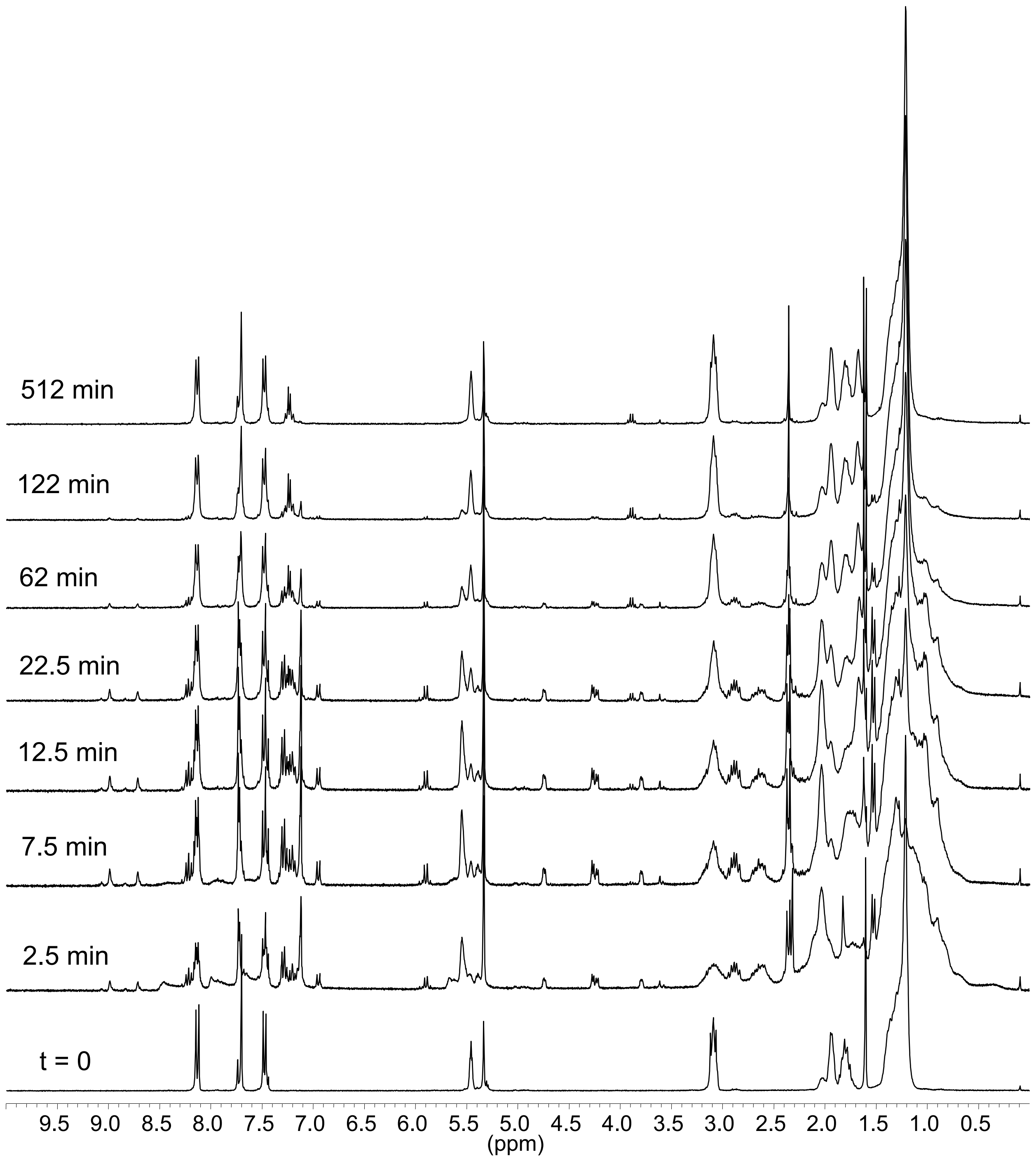

Fig. S7. ^{1}H NMR monitoring of the reaction between 5 mM **1** and 5 mM **2**, X = CH_3 in CD_2Cl_2 at 25 °C.

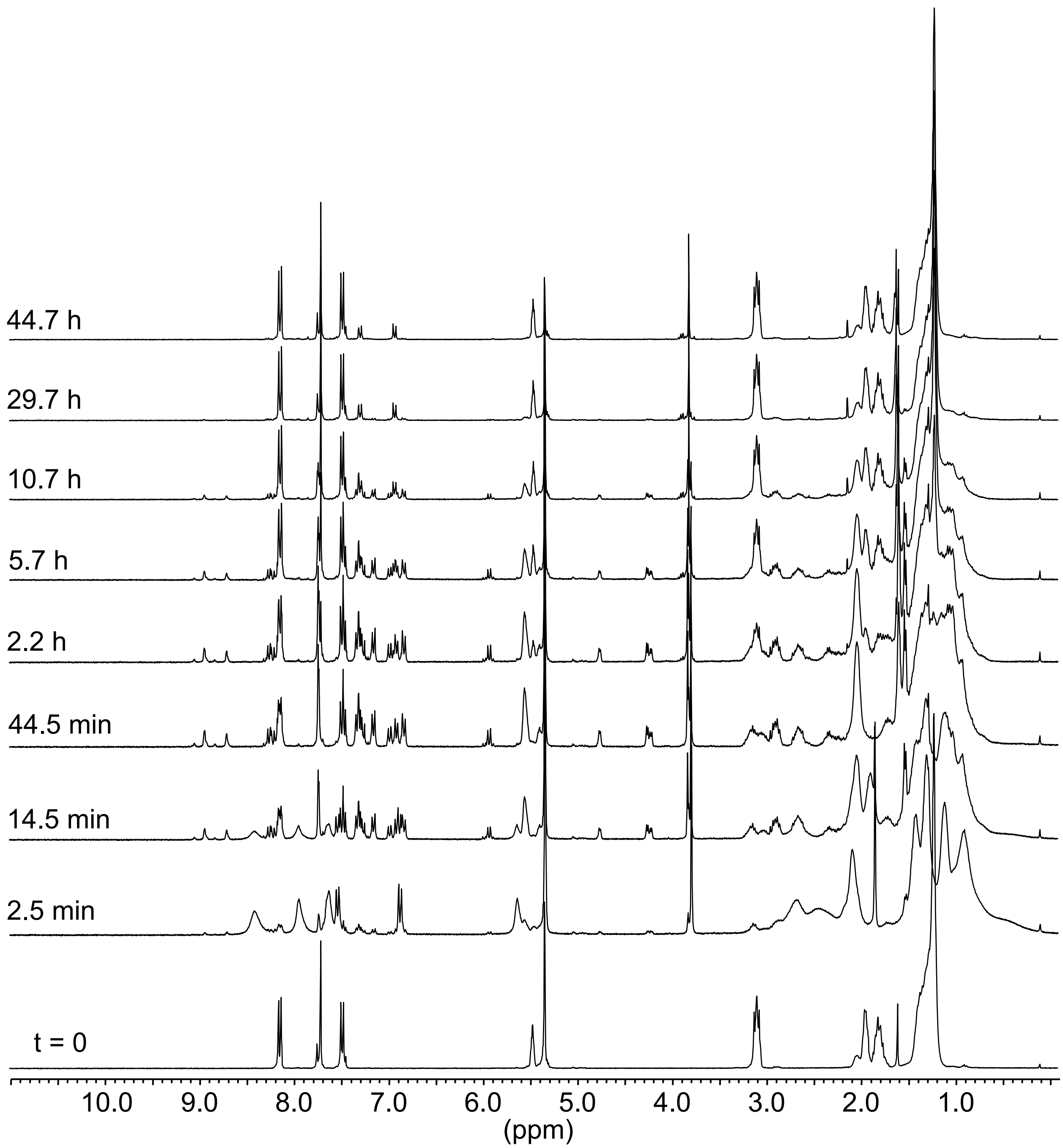

Fig. S8. ^{1}H NMR monitoring of the reaction between 5 mM **1** and 5 mM **2**, X = OCH_3 in CD_2Cl_2 at 25 °C.

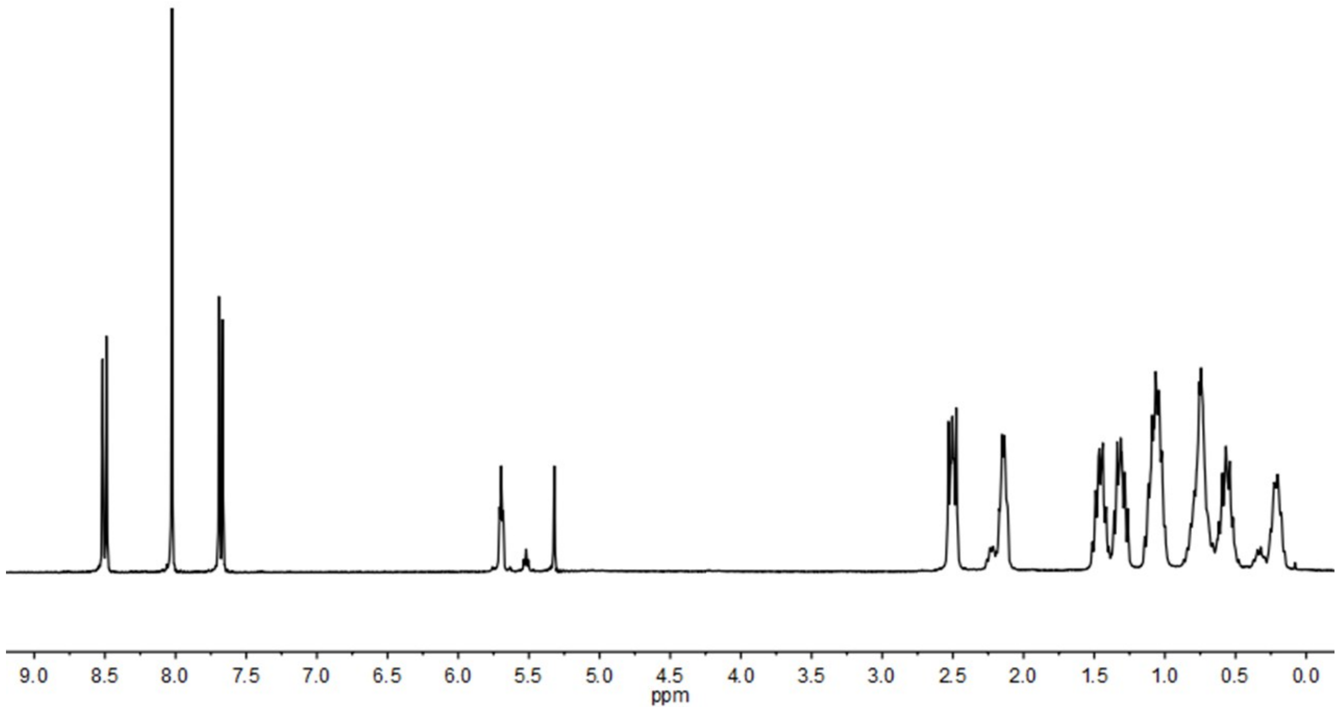

Fig. S9. ^{1}H NMR spectrum of **1**H$^+$ $CF_3CO_2^-$ in CD_2Cl_2 at 25 °C (data from ref 6). Peak at 5.30 is due to $CHDCl_2$.

Consecutive first-order reactions

$$\mathrm{A} \xrightarrow{k'} \mathrm{B}$$

$$\mathrm{B} \xrightarrow{k''} \mathrm{C}$$

The integrated rate eqn (S1)[S1] reduces to the simple form of eqn (S2) when $k' >> k''$.

$$[B] = \frac{[A]_o k'}{k'' - k'}\left(e^{-k't} - e^{-k''t}\right) \qquad \text{(S1)}$$

$$[B] = [A]_o\left(e^{-k''t} - e^{-k't}\right) \qquad \text{(S2)}$$

Eqn (S3) is an adaptation of eqn (S2) to the growth and subsequent decay of absorbance A in the course of the reactions of **1** with acids **2**.

$$A = A_{max}\left(e^{-k''t} - e^{-k't}\right) + A_o e^{-k't} + A_\infty\left(1 - e^{-k''t}\right) \qquad \text{(S3)}$$

A_o and A_∞ are the absorbance values at t = 0 and t = ∞, respectively, and $A_{\max}$ is the maximum value in the absorbance-time profile.

[S1] E. A. Frost and R. G. Pearson, Kinetics and Mechanism, 2nd Edition, John Wiley & Sons, New York 1961.

Effect of reactant concentration on the reaction of **2**, X = OCH_3 with equimolar **1** in CH_2Cl_2 at 25 °C

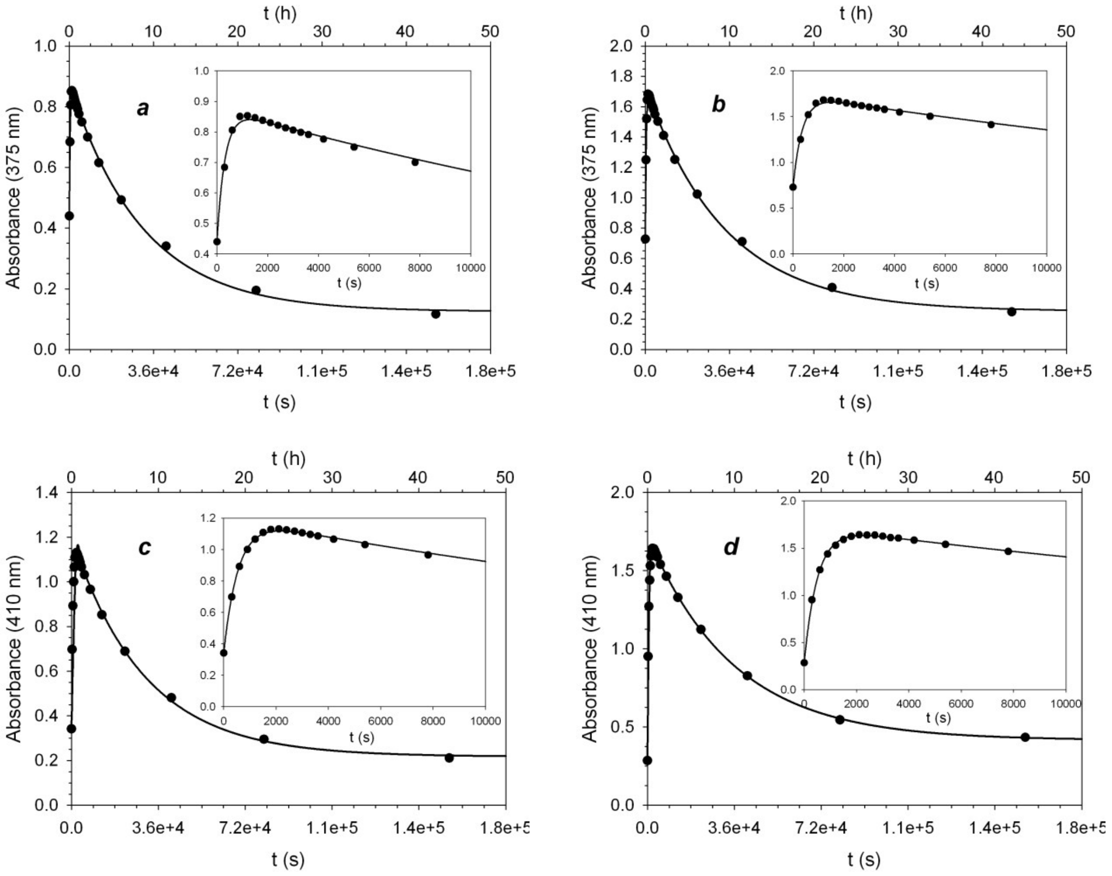

run	conc (mM)	10^3 k' (s^{-1})	10^5 k'' (s^{-1})
a	0.30	3.2	3.2
b	0.60	2.8	3.0
c	1.00	1.9	3.3
d	1.50	2.0	2.8
	Average	2.5±0.5	3.1±0.2

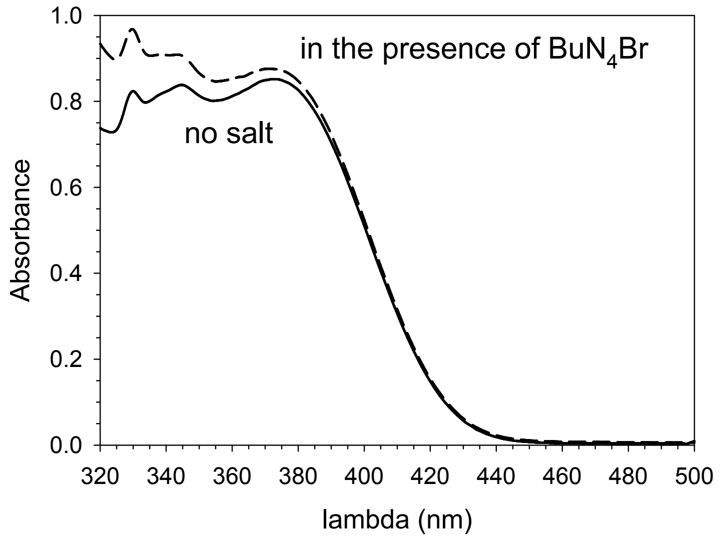

Fig. S10. Reaction between equimolar 0.30 mM **1** and **2**, X = H. Spectrum of the solution at the highest concentration of the intermediate in the absence and in the presence of 5 mM Bu_4NBr

Alternative mechanisms for reaction of 0.30 mM **2**, X = H with equimolar **1** in CH_2Cl_2 at 25 °C in the presence of increasing amounts of Bu_4NBr.

The red curve in Fig. S11 is the plot reported in the main text (Fig. 5) and is related to the association mechanism in Scheme 2

The black curve in Fig. S11 is a plot obtained from the fit of the experimental data points with the system of equations reported below, taking into account the mechanism depicted in Scheme S1. In this case, after a metathetic exchange of counterions (a sort of special salt effect), **1**H^+Br^- and $Bu_4N^+R^-$ react in a bimolecular fashion to give the products.

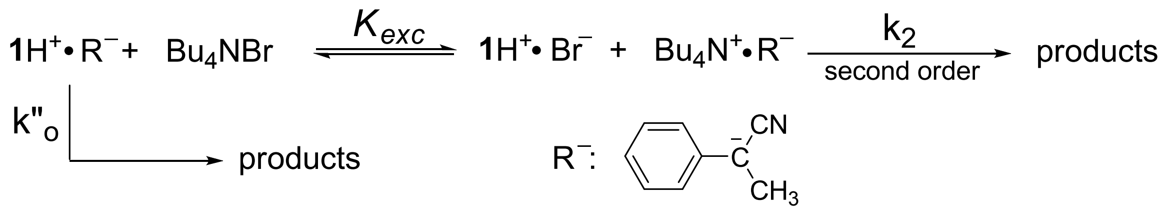

Scheme S1

f=k"$_o$*(((C-z)/C)+k_2*((z^2)/C)
z=(-b-(sqrt((b^2)-4*c*a)))/(2*a)
b=-K_{exc}*(C + x)
a= K_{exc} - 1
c= K_{exc}*C*x
k"$_o$ =2.5e-4 s^{-1}
fit f to y

best fit values
K_{exc} =0.99 ± 0.50
k_2=16.6 ± 2.1 M^{-1} s^{-1}
r = 0.97

The system of equations reported on the left is the one used to obtain the best fit values of K_{exc} and k_2 of Scheme S1 through the calculation software Sigma Plot 10.0.
C is the concentration of reagents **1** and **2**, X = H (3×10^{-4} M);
x is the initial concentration of $Bu_4N^+Br^-$ (ranging from 0 to 5×10^{-3} M);
z is the fraction of intermediate **1**H^+ R^- exchanged with $Bu_4N^+Br^-$.

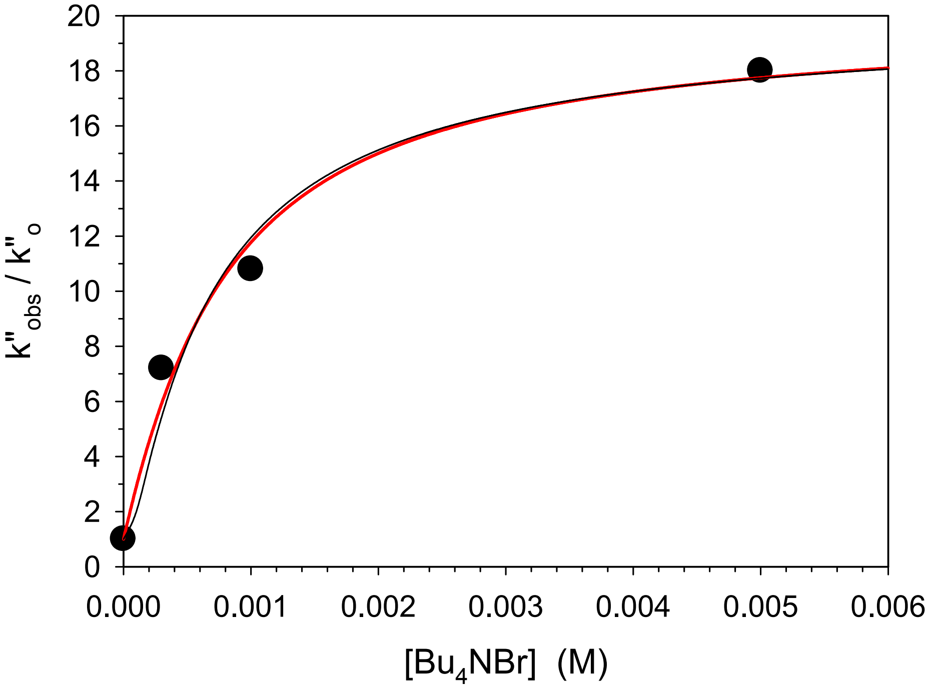

Fig. S11. Alternative fits of the data obtained for reaction of **2**, X = H with equimolar **1** in CH_2Cl_2 at 25 °C in the presence of increasing amounts of Bu_4NBr (in the case of the black fit, $k''_{obs} = k_2 \times z$).
